# PRC2 represses transcribed genes on the imprinted inactive X chromosome in mice

**DOI:** 10.1186/s13059-017-1211-5

**Published:** 2017-05-03

**Authors:** Emily Maclary, Michael Hinten, Clair Harris, Shriya Sethuraman, Srimonta Gayen, Sundeep Kalantry

**Affiliations:** Department of Human Genetics, University of Michigan Medical School, Ann Arbor, MI 48109-5618 USA

**Keywords:** X-inactivation, Polycomb, PRC2, EED, H3K27me3, Imprinting, Trophoblast stem cells

## Abstract

**Background:**

Polycomb repressive complex 2 (PRC2) catalyzes histone H3K27me3, which marks many transcriptionally silent genes throughout the mammalian genome. Although H3K27me3 is associated with silenced gene expression broadly, it remains unclear why some but not other PRC2 target genes require PRC2 and H3K27me3 for silencing.

**Results:**

Here we define the transcriptional and chromatin features that predict which PRC2 target genes require PRC2/H3K27me3 for silencing by interrogating imprinted mouse X-chromosome inactivation. H3K27me3 is enriched at promoters of silenced genes across the inactive X chromosome. To abrogate PRC2 function, we delete the core PRC2 protein EED in F1 hybrid trophoblast stem cells (TSCs), which undergo imprinted inactivation of the paternally inherited X chromosome. *Eed* ^–/–^ TSCs lack H3K27me3 and Xist lncRNA enrichment on the inactive X chromosome. Despite the absence of H3K27me3 and Xist RNA, only a subset of the inactivated X-linked genes is derepressed in *Eed* ^–/–^ TSCs. Unexpectedly, in wild-type (WT) TSCs these genes are transcribed and are enriched for active chromatin hallmarks on the inactive-X, including RNA PolII, H3K27ac, and H3K36me3, but not the bivalent mark H3K4me2. By contrast, PRC2 targets that remain repressed in *Eed* ^–/–^ TSCs are depleted for active chromatin characteristics in WT TSCs.

**Conclusions:**

A comparative analysis of transcriptional and chromatin features of inactive X-linked genes in WT and *Eed* ^–/–^ TSCs suggests that PRC2 acts as a brake to prevent induction of transcribed genes on the inactive X chromosome, a mode of PRC2 function that may apply broadly.

**Electronic supplementary material:**

The online version of this article (doi:10.1186/s13059-017-1211-5) contains supplementary material, which is available to authorized users.

## Background

Post-translational modifications of histones in chromatin are postulated to transmit transcriptional states across cell division [1–3]. Histone H3 trimethylated at lysine 27 (H3K27me3) constitutes a key chromatin modification that is associated with silenced gene expression. H3K27me3 is catalyzed by the Polycomb repressive complex 2 (PRC2), a large evolutionarily conserved multimeric protein complex [4–7]. In mammals, PRC2 and H3K27me3 are implicated in many physiological processes, including pluripotency, differentiation, tumorigenesis, and X-chromosome inactivation [1, 8, 9].

Mammalian PRC2 consists of the core components EZH2, EED, SUZ12, and RBAP48. EZH2 is the enzymatic subunit of PRC2 that catalyzes H3K27me3 [4–7]. EED is required for the stability of PRC2; in *Eed* ^–/–^ cells, EZH2 protein levels are significantly reduced and H3K27me3 is ablated [10]. EED also functions to propagate H3K27me3 at target loci. EED binds to pre-deposited H3K27me3 in S-phase and in turn stimulates EZH2 to catalyze H3K27me3 on newly deposited histones [11, 12]. Thus, EED is required for PRC2 stability, recruitment, and H3K27me3 catalysis.

X-chromosome inactivation has provided essential insights into PRC2 function [8, 13, 14]. X-inactivation is an evolutionarily conserved process that equalizes X-linked gene dosage between *XX* females and *XY* males through the epigenetic silencing of genes on one of the two X chromosomes in females [15, 16]. Two different forms of X-inactivation characterize the early mouse embryo. The initial form is imprinted X-inactivation, in which all cells of the female preimplantation embryo inactivate the paternally inherited X chromosome [17–19]. In later stage embryos, imprinted inactivation of the paternal-X is maintained in the trophectoderm and primitive-endoderm derivatives in the placenta and the yolk-sac, respectively [20, 21]. The epiblast progenitor cells that give rise to the embryo proper, on the other hand, reactivate the paternal-X and subsequently randomly inactivate either the paternal or the maternal X chromosome [17, 22, 23].

At the onset of both imprinted and random X-inactivation, the core PRC2 proteins and H3K27me3 are enriched at transcriptional start sites (TSSs) of genes across the inactive X chromosome [24–27]. PRC2 is proposed to be recruited to the inactive X chromosome, either directly or indirectly, by the Xist long non-coding (lnc) RNA [28–30], which is transcribed only from the inactive X chromosome and is necessary for stable X-inactivation [31–33]. By virtue of its early enrichment on the inactive-X and its gene silencing function, PRC2 is thought to be essential for X-inactivation [26, 27]. In agreement, the extra-embryonic tissues in female *Eed* ^–/–^ mouse embryos and differentiating *Eed* ^–/–^ trophoblast stem cells (TSCs) display defects in imprinted X-inactivation [27, 34, 35]. Although PRC2 and H3K27me3 are enriched at silenced genes across the inactive X chromosome, it remains unclear if some or all genes on the inactive-X require PRC2 for silencing.

In this study, we generated F1 hybrid wild-type (WT) and *Eed* ^–/–^ TSC lines harboring polymorphic X chromosomes, thus enabling a comprehensive analysis of gene expression and chromatin states on the inactive X chromosome. *Eed* ^–/–^ TSCs lack H3K27me3 and Xist RNA expression and coating. Despite the absence of H3K27me3 and Xist RNA coating, however, we find that fewer than one-fifth of the X-linked genes are significantly derepressed from the inactive paternal X chromosome in *Eed* ^–/–^ TSCs. Profiles of the X-chromosome transcriptome, conformation, and evolutionary history coupled with the assessment of DNAseI hypersensitivity, RNA PolII occupancy, and a panel of histone modifications demonstrate that the genes that are derepressed in *Eed* ^–/–^ TSCs are unexpectedly transcribed and are enriched for select hallmarks of open chromatin on the imprinted inactive X chromosome in WT TSCs. X-linked genes that are stringently silenced in WT TSCs, however, are refractory to the loss of EED, H3K27me3, and Xist RNA, and remain repressed in *Eed* ^–/–^ TSCs. Thus, transcriptional activity predicts which genes on the imprinted inactive X chromosome require PRC2 for silencing.

## Results

### Loss of H3K27me3 enrichment and Xist RNA coating on the inactive X chromosome in *Eed*^–/–^ TSCs

**Fig. 1:**
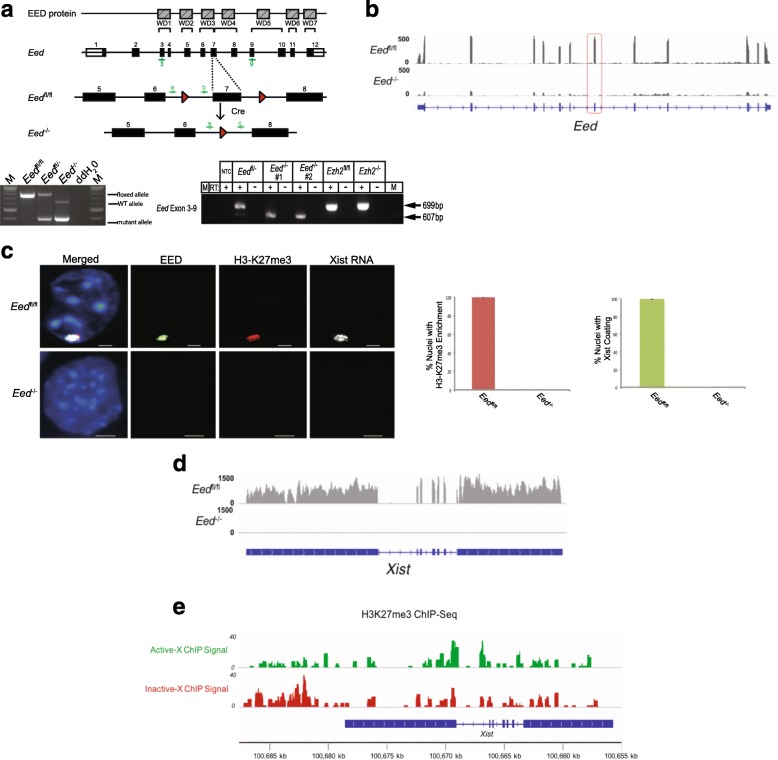
Absence of H3K27me3 enrichment and Xist RNA coating on the inactive X chromosome in *Eed* ^–/–^ TSCs. **a** *Top*, *diagram* of conditional *Eed* mutation. *Bottom left*, *gel image* of genotyping PCR. *Bottom right*, RT-PCR detection of *Eed* RNA in *Eed* ^fl/–^, *Eed* ^–/–^, and *Eed* ^+/+^ samples. The location of PCR and RT-PCR primers are depicted in the diagram above in *blue* and *green*, respectively. **b** Loss of *Eed* exon 7 and reduction of mRNA in *Eed* ^–/–^ line by RNA-seq. **c** Detection of EED (*green*) and H3K27me3 (*red*) by immunofluorescence (IF) and Xist RNA (*white*) by RNA FISH in *Eed* ^fl/fl^ and *Eed* ^–/–^ TSCs. Nuclei are stained *blue* with DAPI. Scale bar, 2 μm. *Right*, quantifications of numbers of nuclei with H3K27me3 enrichment and Xist RNA coating in *Eed* ^fl/fl^ and *Eed* ^–/–^ TSCs. **d** Absence of Xist RNA expression in *Eed* ^–/–^ TSCs by RNA-seq. **e** Enrichment of H3K27me3 at Xist promoter region on the inactive paternal-X in WT TSCs

We generated an F1 hybrid female *Eed* ^fl/fl^ TSC line from a conditionally mutant *Eed* mouse strain in which an exon encoding part of one of the evolutionarily conserved WD40 repeats (WD3) is flanked by *loxP* sequences (Fig. 1a). EED WD40 domains have been shown to be necessary for EED to interact with EZH2, the PRC2 enzyme [36]. Therefore, a perturbation of the WD40 motifs in TSCs is predicted to disrupt essential PRC2 interactions and function, effectively creating a null mutation. We subsequently isolated three *Eed* ^–/–^ TSC subclones by transducing *Eed* ^fl/fl^ TSCs with a Cre recombinase-expressing adenovirus (Fig. 1a). RNA sequencing (RNA-seq) confirmed loss of *Eed* exon 7 and a reduction in Eed messenger RNA (mRNA) level in *Eed* ^–/–^ TSC lines (Fig. 1b).

We assessed enrichment of EED protein and H3K27me3 on the inactive X chromosome in *Eed* ^fl/fl^ and *Eed* ^–/–^ TSC lines by immunofluorescence (IF). In the same cells, we also evaluated Xist RNA expression and localization by fluorescent in situ hybridization (FISH), since Xist RNA accumulation marks the inactive X chromosome [37, 38]. As expected, EED and H3K27me3 were enriched on the Xist RNA-coated inactive paternal X chromosome in *Eed* ^fl/fl^ cells. *Eed* ^–/–^ TSCs, by contrast, lacked EED and H3K27me3 enrichment as well as Xist RNA coating (Fig. 1c, Additional file 1: Figures S1a, b). RNA-seq and quantitative reverse transcription polymerase chain reaction (RT-PCR) demonstrated that Xist RNA expression was absent in *Eed* ^–/–^ TSCs (Fig. 1d, Additional file 1: Figure S1c). Expression of Tsix RNA, the antisense regulator of *Xist*, varies between cell lines but is not altered by EED loss (Additional file 1: Figure S1c). Thus, loss of EED in TSCs results in the corresponding loss of H3K27me3 and Xist RNA coating of the paternal X chromosome that is subject to imprinted X-inactivation, consistent with previous data [35].

The loss of Xist RNA coating in the *Eed* ^–/–^ TSCs suggests a role for H3K27me3 in inducing Xist. H3K27me3 transiently accumulates at the *Xist* locus at the onset of Xist expression in differentiating female embryonic stem cells (ESCs) [28]. To examine deposition of H3K27me3 at the Xist promoter on the inactive-X in TSCs, we interrogated allele-specific H3K27me3 chromatin immunoprecipitation sequencing (ChIP-seq) data from F1 hybrid WT TSCs [39]. H3K27me3 is in fact enriched upstream of the *Xist* TSS on the inactive paternal X chromosome in TSCs (Fig. 1e, Additional file 1: Figure S1d).

### RNA FISH reveals limited derepression of X-linked genes in *Eed*^–/–^ TSCs

**Fig. 2:**
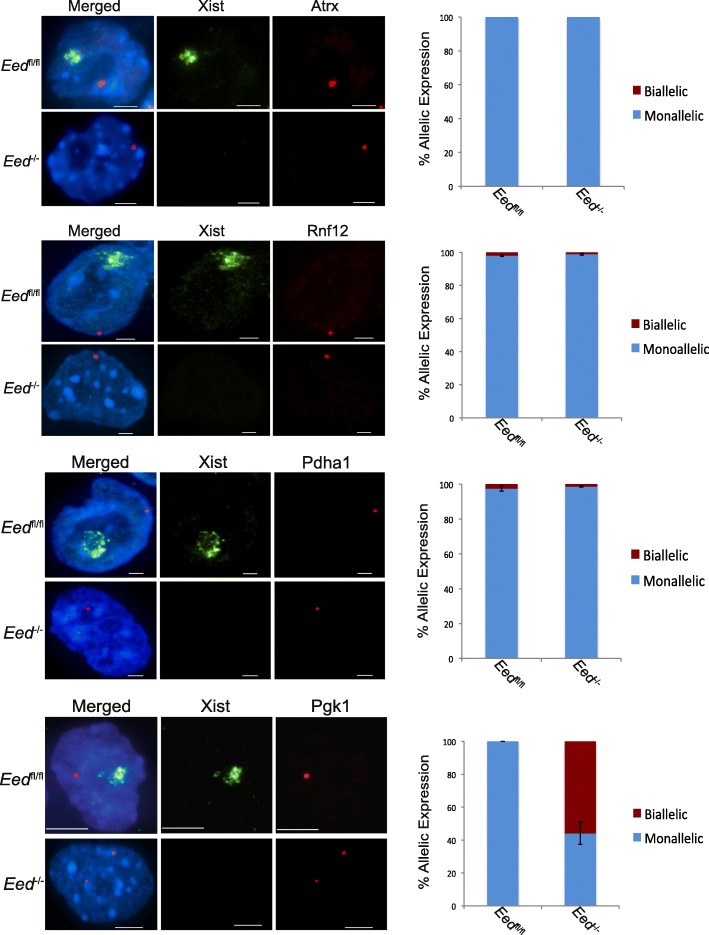
RNA FISH analysis of X-linked gene expression in *Eed* ^–/–^ TSCs. *Left*, RNA FISH detection of Xist RNA coating (*green*) and RNAs of four X-linked gene (*Atrx*, *Rnf12*, *Pdha1*, and *Pgk1*, *red*) in *Eed* ^fl/fl^ and *Eed* ^–/–^ TSCs. Nuclei are stained *blue* with DAPI. Scale bar, 2 μm. *Right*, quantifications of the RNA FISH data. Whereas *Atrx*, *Rnf12*, and *Pdha1* remain silenced in *Eed* ^–/–^ TSCs, *Pgk1* is derepressed from the inactive-X in the absence of EED. Three technical replicates of the *Eed* ^fl/fl^ TSC line and three different *Eed* ^–/–^ TSC lines were stained; 100 nuclei counted/gene/TSC sample

To test whether X-inactivation is compromised in *Eed* ^–/–^ TSCs, we assayed the expression of four X-linked genes subject to X-inactivation, *Atrx*, *Rnf12*, *Pdha1*, and *Pgk1*, by RNA FISH. Both the parental *Eed* ^fl/fl^ TSC line and the three derived *Eed* ^–/–^ female TSC lines displayed monoallelic expression of *Atrx*, *Rnf12*, and *Pdha1* (Fig. 2). In *Eed* ^–/–^ TSCs, all three genes appeared to be silenced on one allele, presumptively on the inactive-X, despite the absence of H3K27me3 and Xist RNA coating. The fourth X-linked gene, *Pgk1*, however, was monoallelically expressed in *Eed* ^fl/fl^ TSCs but biallelically expressed in *Eed* ^–/–^ TSCs (Fig. 2). To validate these data, we generated an independent *Eed* ^–/–^ TSC line from a non-F1 hybrid *Eed* ^fl/fl^ TSC line harboring an inducibly expressed *Cre* transgene (*Cre-ERT2*). This *Eed* ^–/–^ TSC line also lacked Xist RNA expression and coating and also displayed monoallelic expression of *Atrx*, *Rnf12*, and *Pdha1* but biallelic expression of *Pgk1* (Additional file 1: Figure S1e). Together, these results imply that some genes, but not others, are subject to derepression on the paternal X chromosome in *Eed* ^–/–^ TSCs.

### X-chromosome-wide identification of derepressed genes in *Eed*^*–/–*^ TSCs

**Fig. 3:**
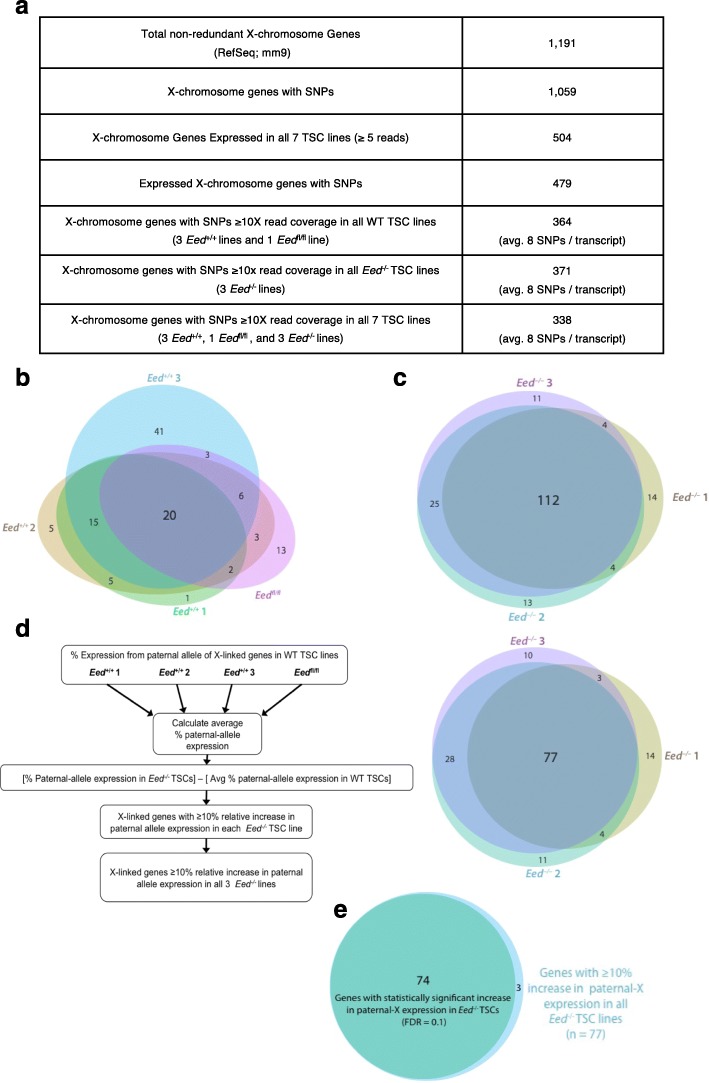
Identification and characterization of paternal-X expression in *Eed* ^*+/+*^, *Eed* ^fl/fl^, and *Eed* ^–/–^ TSCs. **a** Summary of allele-specific RNA-seq analysis of X-linked genes. **b**, **c** Euler diagrams assessing expression from the paternal X chromosome totaling ≥ 10% of total expression in three WT *Eed* ^*+/+*^ TSC lines and one WT *Eed* ^fl/fl^ TSC line (**b**) and in three *Eed* ^–/–^ TSC lines (**c**). **d** Comparison of percent paternal-X expression in *Eed* ^–/–^ TSC lines compared with WT TSC lines. Percent paternal-X expression for individual *Eed* ^–/–^ TSC lines was compared with the average percent paternal-X expression in WT TSC lines (*left*). **e** Identification of genes with statistically significant difference in percent paternal-X expression in *Eed* ^–/–^ TSCs compared with WT TSCs. Percent paternal-X expression in the four WT TSC lines and three *Eed* ^–/–^ TSC lines was compared by T-test for the 77 genes that display a consistent increase in paternal-X expression in *Eed* ^–/–^ TSC lines. Of these, 74 genes are statistically significantly increased in paternal-X expression in *Eed* ^–/–^ TSC lines, following Benjamini–Hochberg correction of the *p* values for multiple testing (FDR = 0.1)

To comprehensively identify inactive X-linked genes that are derepressed in *Eed* ^–/–^ TSCs, we performed RNA-seq on the F1 hybrid control *Eed* ^*+/+*^ and *Eed* ^fl/fl^ (both classified as WT genotype hereafter) and test *Eed* ^–/–^ female TSCs. The polymorphic X chromosomes in the hybrid TSCs allowed for allele-specific analysis of X-linked gene expression. Whereas the maternal X chromosome was derived from the *Mus musculus* 129/S1 mouse strain, the paternal-X originated from the *Mus molossinus* JF1/Ms strain. The genomes of the 129/S1 and JF1/Ms strains are highly divergent and contain many defined single nucleotide polymorphisms (SNPs) [40–42]. Since TSCs undergo imprinted X-inactivation [43], the 129/S1-derived maternal X chromosome is the active-X and the JF1/Ms-derived paternal X chromosome is the inactive-X. We were thus able to exploit the strain-specific SNPs to profile expression of genes across the active and inactive X chromosomes (see details in “Methods”). From the RefSeq mm9 genome annotation, 89% of the non-redundant set of X-linked genes contained at least one SNP (Fig. 3a). For all SNP sites with ≥ 10X read coverage, we calculated the percent paternal-X expression based on the fraction of SNP-overlapping reads that mapped to the paternal (JF1/Ms) versus the maternal (129/S1) allele (see details in “Methods” and example in Additional file 1: Figure S2). We then averaged percent paternal allele expression at all SNP sites that reached the 10X read coverage threshold within a given X-linked gene, to calculate the percent inactive-X expression of individual genes.

We uncovered 364 and 371 X-linked genes in the four WT TSC lines and three *Eed* ^–/–^ TSC lines, respectively, that satisfied the 10X coverage threshold at SNP sites (Fig. 3a; Additional file 2: Table S1; Additional file 3: Table S2). A common set of 338 genes reached the coverage threshold at SNP sites in all seven WT and *Eed* ^–/–^ TSC lines. In all seven datasets, most transcripts harbored multiple SNPs reaching the 10X coverage threshold, with an average of eight SNPs per transcript (Fig. 3a). The RNA-seq data recapitulated the expression patterns of the X-linked genes surveyed by RNA FISH in Fig. 2 (Additional file 2: Table S1).

Genes with ≥ 10% of total expression from the paternal X chromosome have previously been classified as X-inactivation escapees [44–47]. Using this threshold, 20 of the 364 expressed X-linked genes (5%) escaped X-inactivation in all four WT TSC lines (Fig. 3b). This set included genes previously shown to escape X-inactivation such as *Kdm5c* and *Eif2s3x* [39, 44, 47, 48]. Also included are genes that escape X-inactivation in TSCs but not in other tissues, such as *Yipf6* [39]. An additional 12 genes not previously reported to escape X-inactivation displayed ≥ 10% of total expression from the paternal X chromosome in the WT TSC lines; these unique escapers include genes such as *Dusp9* and *Atp7a* (see Additional file 3: Table S2 for complete list). Cell type-specific and strain-specific differences may explain why these genes have previously not been identified as escapees.

We next interrogated allele-specific X-linked gene expression in *Eed* ^–/–^ TSCs. The distribution of paternal-X expression for SNP-containing genes in *Eed* ^–/–^ TSCs differs from those of WT TSC lines. Compared with the WT TSCs, a substantially higher number of genes showed ≥ 10% paternal allele expression in all three *Eed* ^–/–^ TSCs (112/371 or 30%) (Fig. 3c; see also Additional file 1: Figure S3 and Additional file 3: Table S2). Contained within this subset are 18 of the 20 genes that escape X-inactivation in the four WT TSCs. The two escapees unique to WT TSCs do not reach the required 10X depth of coverage at SNP sites in *Eed* ^–/–^ TSCs, but are nevertheless expressed from the paternal-X in the mutant cells (Additional file 3: Table S2). An additional 94 genes that are subject to X-inactivation in the WT TSCs were expressed from the paternal X chromosome in the three *Eed* ^–/–^ TSCs. Overall, the percentage of expressed paternal X-linked genes was much higher in *Eed* ^–/–^ TSCs (30%) compared with WT TSCs (5%).

We next calculated the extent of change in expression of paternal X-linked genes in *Eed* ^–/–^ versus WT TSCs. We compared the ratio of paternal to total (paternal + maternal) allelic expression for the common set of 338 X-linked genes with ≥ 10X SNP-overlapping read coverage in the three *Eed* ^–/–^ TSC lines to the percent paternal allele expression averaged across the four WT TSC lines (Fig. 3d). Applying the threshold used to delineate X-inactivation escapees in WT TSCs, we defined X-linked genes whose relative paternal allele expression increased by ≥ 10% in *Eed* ^–/–^ TSCs compared with the average percent paternal allele expression in WT TSCs as candidates that are derepressed from the inactive X chromosome in the absence of EED. A total of 77 of the 338 genes, or 23%, reached this threshold. Seventy-two of the 77 genes are subject to X-inactivation in WT TSCs. The remaining five genes escaped X-inactivation in the WT TSCs, but were nevertheless increased by ≥ 10% from the paternal allele in *Eed* ^–/–^ TSCs. Of the set of 77 genes that demonstrated an increase in expression from the paternal-X in all three *Eed* ^–/–^ TSC lines, 74 displayed a statistically significant difference between the proportion of paternal-X expression in *Eed* ^–/–^ TSC lines versus the control WT TSC lines (Welch’s two-sample T-test followed by Benjamini–Hochberg correction for multiple testing; FDR = 0.1) (Fig. 3e; Additional file 3: Table S2).

**Fig. 4:**
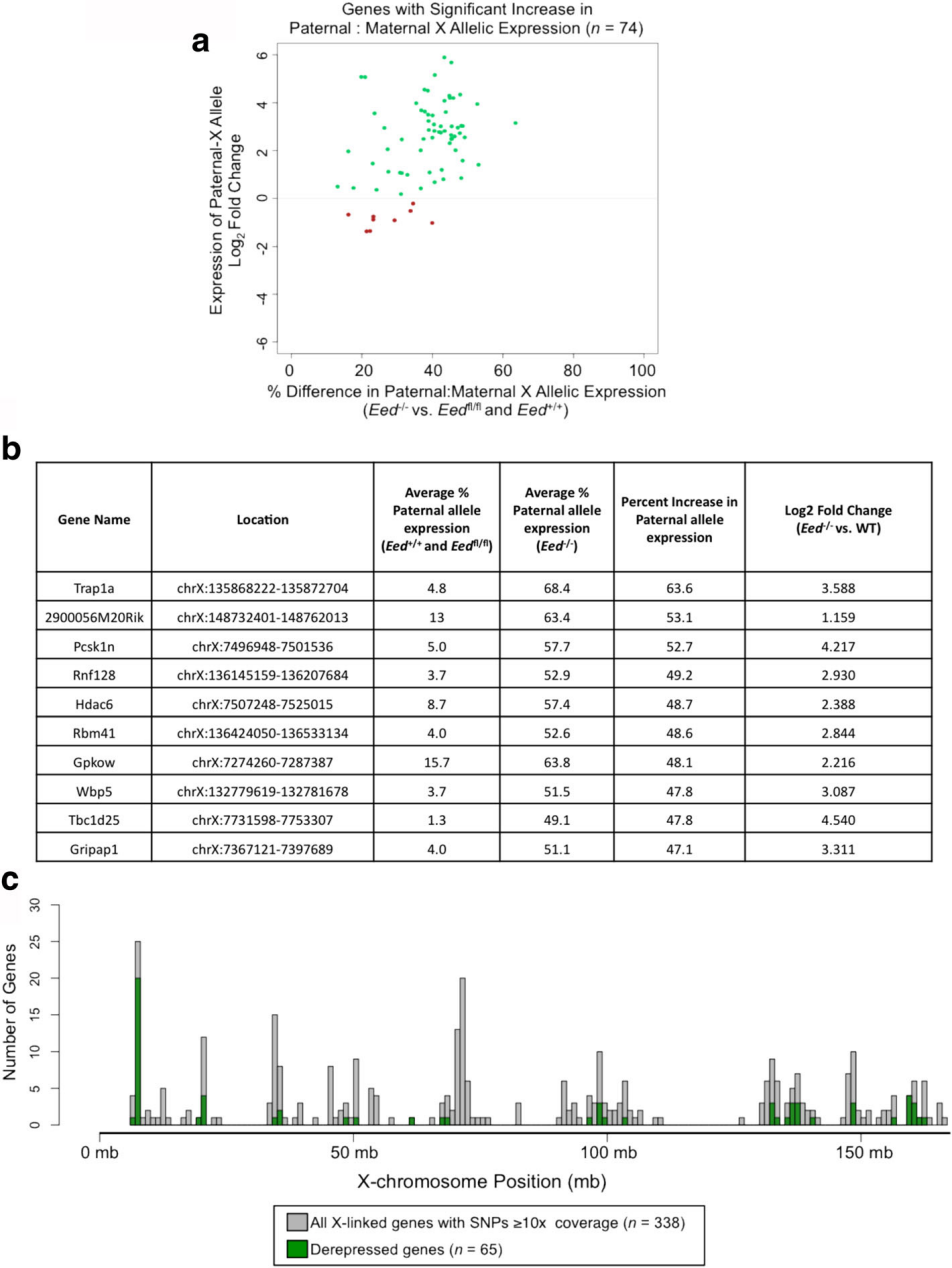
Characterization of expression and chromosomal location of genes with increased paternal-X expression in *Eed* ^–/–^ TSCs. **a** *Plot* of log_2_ fold change between genotypes in the expression of paternal allele, as calculated by DESeq2 (*y-axis*) vs. percent difference in paternal allele expression between *Eed* ^–/–^ and WT TSCs (*x-axis*) for the 74 X-linked genes exhibiting a statistically significant increase in the relative proportion of paternal:maternal allele expression. Sixty-five genes are upregulated (*green*) and nine genes are downregulated (*red*) from the paternal-X. **b** *Table* summarizing the ten X-linked genes with the greatest increase in paternal allele expression in *Eed* ^–/–^ TSCs. **c** *Histogram* indicating the location of all 338 X-linked genes with informative SNPs with ≥ 10X coverage (*gray bars*) and the 65 upregulated genes whose relative expression from the paternal allele is significantly increased (*green bars*). Derepressed X-linked genes are distributed across the length of the X chromosome

In principle, the relative increase in expression from the paternal-X allele in *Eed* ^–/–^ TSCs could be driven by one of two major mechanisms: either increased expression of the paternal-X allele or reduced expression from the maternal-X allele. In order to distinguish between these two possibilities, we performed differential analysis of paternal X-linked gene expression between WT and *Eed* ^–/–^ TSCs [49]. For genes whose shift in allelic expression is driven by the derepression of the paternal-X allele, both the relative proportion of total expression from the paternal allele as well as the absolute expression from the paternal allele are expected to increase. SNP-containing reads may only cover a fraction of a given gene, thus they alone cannot be used for differential expression analysis. We therefore calculated read coverage of the entire gene, including reads overlapping both polymorphic and non-polymorphic sites. From the total read counts, we calculated paternal-X expression by multiplying the number of mapped reads for each gene by the proportion of SNP-containing reads mapping to the paternal X chromosome (see detailed description in “Methods”). We then performed differential expression analysis on these calculated paternal-X expression levels between the four WT TSC lines and the three *Eed* ^–/–^ TSC lines (Additional file 4: Table S3; Fig. 4a). The majority of X-linked genes exhibiting a relative increase in expression from the paternal allele also showed an increase in the absolute expression from the paternal X chromosome (65/74, or 85%, green dots in Fig. 4a). The remaining nine genes displayed either negligible changes or a decrease in the absolute level of paternal-X expression. In these genes, the observed shift in the proportion of paternal allele expression is likely due to the downregulation of the maternal allele.

We therefore defined paternally derepressed genes as the set of 65 X-linked genes (19% of the 338 genes assayed) that exhibit both relative and absolute upregulation of the paternal allele in *Eed* ^–/–^ TSCs compared with WT TSCs (Additional file 3: Table S2; Additional file 4: Table S3). The ten highest derepressed X-linked genes in *Eed* ^–/–^ TSCs are summarized in Fig. 4b. To confirm that the gene-specific derepression observed is not simply a function of the thresholds set for read coverage and allelic balance, we additionally assessed expression from the paternal X chromosome in all TSC lines at a less stringent threshold, requiring only 5X coverage of SNPs. The lower stringency criteria yielded derepression of a similar subset of paternal X-linked genes in *Eed* ^–/–^ TSCs (21%) (Additional file 1: Figure S4; Additional file 5: Table S4).

We next validated the allele-specific RNA-seq data by RT-PCR followed by Sanger sequencing. We selected six genes distributed across the X chromosome, *Hdac6*, *Wdr13*, *Med12*, *Pgk1*, *Wbp5*, and *Rnf128*, identified as derepressed from the paternal X chromosome in *Eed* ^–/–^ TSCs by RNA-seq (Additional file 1: Figure S5a). We also chose six X-linked genes, *C330007P06Rik*, *Atp11c*, *Fam3a*, *Rnf12*, *Atrx*, and *Pdha1*, that stably maintain silencing of the paternal allele in *Eed* ^–/–^ TSCs by RNA-seq (Additional file 1: Figure S5a). For each gene, we designed amplicons spanning SNPs between the 129/S1 and JF1/Ms mouse strains (see Additional file 6: Table S5 for primer and SNP information). For all six genes found to be derepressed in *Eed* ^–/–^ TSCs by RNA-seq, the Sanger sequencing chromatograms exhibited expression from only the maternal (active)-X allele in all four WT TSC lines but biallelic expression in all three *Eed* ^–/–^ TSC lines (Additional file 1: Figure S5b). For the six X-linked genes that did not display an allelic shift in the RNA-seq data, the WT as well as the *Eed* ^–/–^ TSCs displayed expression from only the maternal allele (Additional file 1: Figure S5c). We also validated the presence of the genomic loci of the genes assayed by RT-PCR. Sanger sequencing of PCR-amplified genomic DNAs from WT and *Eed* ^–/–^ TSC lines, using amplicons spanning the same polymorphic sites assayed by RT-PCR, demonstrated that both alleles of all the genes tested were intact in all TSC lines (Additional file 1: Figure S5d, e).

### Chromosome topology does not determine derepression of paternal X-linked genes upon EED loss

We next sought to determine if the derepressed X-linked genes in *Eed* ^–/–^ TSCs shared any unifying features in WT TSCs, which may predict their derepression upon EED loss. We first tested if the derepressed genes were clustered in specific regions of the X chromosome, which may suggest that those regions are particularly sensitive to loss of EED, H3K27me3, or Xist RNA coating. The derepressed genes were distributed along the length of the X chromosome and did not occupy any one specific region (Fig. 4c). A subset of the derepressed genes, however, was clustered in a gene-dense region within qA1.1 and in a few other regions of the X chromosome. We reasoned that the three-dimensional (3D) structure of the X chromosome might explain such non-linear arrangement of the clusters and took advantage of published allele-specific Hi-C data from female mouse ESCs [50, 51]. The allele-specific Hi-C data identify topologically associated domains (TADs) on the active and inactive X chromosomes. In mouse cells, the active-X forms a series of well-defined TADs. The inactive-X, on the other hand, is structured into two large superdomains that meet at a “hinge” region centered on the *Dxz4* minisatellite repeat in both mouse and human [52, 53], rather than into the discrete TADs that characterize the active-X [50, 52–54]. When Xist RNA is knocked down in X-inactivated cells, the inactive X chromosome now takes on a conformation more similar to the active-X [50]. We therefore tested whether the derepressed X-linked genes in *Eed* ^–/–^ TSCs occupied regions that correspond to specific TADs on the active X chromosome. The spike of derepressed genes within qA1.1 indeed occupies a single TAD on the active X chromosome in WT ESCs and in X-inactivated cells in which Xist RNA is knocked down. Nevertheless, many derepressed X-linked genes do not appear to cluster in specific TADs or lie in or near defined TAD boundaries (Additional file 1: Figure S6a).

We next tested if the period during evolution when the derepressed X-linked genes underwent dosage compensation predicts their derepression. The shared evolutionary history may make the X-linked genes similarly dependent on H3K27me3 and Xist RNA coating for silencing [33, 55, 56]. Prior work has delineated five distinct evolutionary strata on the X chromosome and genes within each stratum are thought to have been subjected to dosage compensation as a unit [57–59]. The evolutionary strata on the human X chromosome are collinear but have been rearranged on the mouse X chromosome [60]; thus, a shared evolutionary history could explain the non-linear arrangement of derepressed X-linked genes in *Eed* ^–/–^ TSCs. We found, however, that derepressed genes did not belong to a single evolutionary stratum (Additional file 1: Figure S6b).

Xist RNA expression is lost in *Eed* ^–/–^ TSCs; the derepression of X-linked genes may thus reflect increased dependency on Xist RNA for silencing. We therefore next tested whether the set of 65 significantly derepressed X-linked genes displayed increased Xist RNA binding in WT cells. Interrogating previously published CHART-seq (capture hybridization analysis of RNA targets with deep sequencing) data assessing where Xist RNA is bound on the inactive-X in ESCs [61], we observed similar enrichment of Xist RNA at X-linked genes that were derepressed versus genes that remained silenced in *Eed* ^–/–^ TSCs (Additional file 1: Figure S7). Thus, differential Xist RNA binding did not appear to influence derepression of X-linked genes upon EED loss.

### Derepressed X-linked genes display active/open chromatin architecture on the inactive-X in WT TSCs

To determine if specific chromatin characteristics may predict the derepression of X-linked genes in *Eed* ^–/–^ TSCs, we next interrogated a panel of chromatin modifications and indicators of chromatin structure from F1 hybrid WT TSCs [39]. We examined chromatin features on the inactive-X in WT TSCs for the set of 65 genes that were derepressed in *Eed* ^–/–^ TSCs, the set of 253 genes that remained silenced, and the set of 20 genes that escape X-inactivation.

**Fig. 5:**
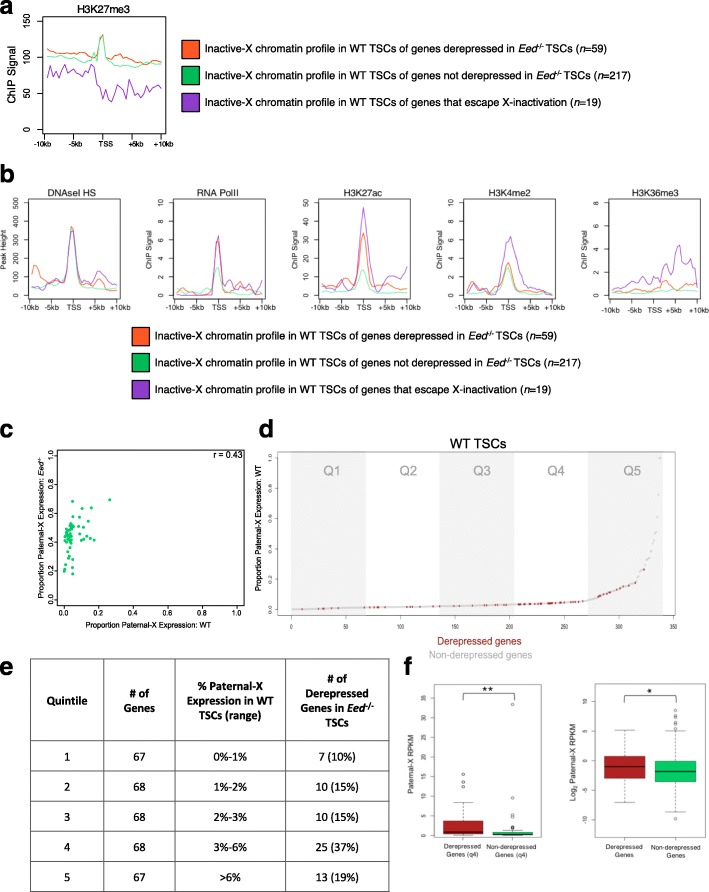
Derepressed genes are transcribed and enriched for active chromatin marks in WT TSCs. **a** Inactive-X profiles of H3K27me3 in WT TSCs of derepressed and non-derepressed genes in *Eed* ^–/–^ TSCs and of X-inactivation escapees in WT TSCs. **b** Inactive-X profiles for DNAseI hypersensitivity (DNAseI HS), RNA PolII occupancy, H3K27ac, H3K4me2, and H3K36me3. **c** Correlation between percent paternal-X expression in WT and *Eed* ^–/–^ TSCs. **d** Paternal-X contribution for all expressed X-linked genes in WT TSCs. The *x-axis* indicates the rank order of each gene from least to most paternal-X expression in WT TSCs. *Gray dots* indicate non-derepressed genes and *red dots* mark genes that are derepressed in *Eed* ^–/–^ TSCs. The *y-axis* depicts percent paternal-X expression for each gene. The expressed paternal X-linked genes can be divided into five quintiles. The derepressed genes are over-represented in quintile 4. **e** *Table* of number of genes and their percent paternal-X expression within the quintiles described in (**d**). **f** *Left*, *boxplots* of absolute paternal-X expression (as RNA-seq reads per kilobase of gene per million reads [RPKM]) for non-derepressed and derepressed genes in quintile 4 (3–6% paternal-X:total X-chromosomal expression). Derepressed genes exhibit a significantly higher median expression level than non-derepressed genes (*p* < 0.001, Mann–Whitney U test). *Right*, *boxplots* of absolute paternal-X expression (Log_2_ scaled, as RPKM) for all non-derepressed and derepressed genes. As with quintile 4, derepressed genes exhibit a significantly higher median expression level than non-derepressed genes (*p* < 0.05, Mann–Whitney U test)

Due to the essential role of EED in H3K27me3 catalysis and the proposed silencing function of H3K27me3 [10], we first tested whether genes that are sensitive to EED loss are normally occupied by higher levels of H3K27me3 compared with genes that are refractory to EED absence. Consistent with the accumulation of H3K27me3 on the inactive X chromosome detected cytologically, H3K27me3 was enriched at the TSSs of paternal X-linked genes in WT TSCs by ChIP-seq. Notably, in WT TSCs H3K27me3 is not present at higher levels at the TSSs of inactive X-linked genes that are derepressed compared with genes that remain silenced in *Eed* ^–/–^ TSCs, suggesting that H3K27me3 levels do not predict susceptibility to reactivation (Fig. 5a). In contrast to H3K27me3 enrichment on the X-inactivated genes, TSSs of genes that escape X-inactivation displayed a reduced average H3K27me3 level in the WT TSCs.

We next analyzed DNAseI hypersensitivity, which characterizes nucleosome-free DNA and is a feature of regulatory elements and TSSs of active genes [62]. In WT TSCs, DNAseI hypersensitivity at the TSSs of genes on the inactive-X that are derepressed in *Eed* ^–/–^ TSCs did not differ from non-derepressed genes (Fig. 5b; Additional file 1: Figure S8a–c). Surprisingly, however, in WT TSCs RNA PolII displayed a trend toward increased occupancy at X-linked genes that are derepressed versus non-derepressed in *Eed* ^–/–^ TSCs (Fig. 5b; Additional file 1: Figure S8a–c). Similarly, in F1 WT TSCs H3K27 acetylation (H3K27ac), which marks loci with open chromatin, and H3K36me3, which marks active transcription, were enriched at TSSs and in the gene body, respectively, in genes that are derepressed compared with non-derepressed in *Eed* ^–/–^ TSCs (Fig. 5b; Additional file 1: Figure S8a–c). H3K4me2, which is also associated with active transcription, however, did not markedly differ between derepressed and non-derepressed genes (Fig. 5b; Additional file 1: Figures S8a–c and S9).

### X-linked genes derepressed in *Eed*^–/–^ TSCs are expressed at low levels from the inactive-X in WT TSCs

Genes with open chromatin architecture are predicted to be expressed. However, high H3K27me3 occupancy at TSSs may argue against productive transcription. We therefore examined if the X-linked genes that are derepressed in *Eed* ^–/–^ TSCs are transcriptionally active on the otherwise inactive X chromosome in WT TSCs. Consistent with published datasets [39], we found that transcription from the paternal-X in TSCs occurs on a broad and highly variable spectrum. Although few genes displayed ≥ 10% expression from the paternal-X in WT TSCs (i.e. the X-inactivation escapees), many showed a low but detectable level of paternal-X expression, in the range of 1–10% of total expression.

To determine if in WT TSCs genes that are derepressed in *Eed* ^–/–^ TSCs displayed higher average levels of transcription from the paternal X chromosome than genes that remain silenced, we first evaluated the relationship between percent paternal-X expression in WT and *Eed* ^*–/–*^ TSCs, which showed a weak positive correlation (*r* = 0.43; Fig. 5c). This correlation suggested that the genes with higher paternal-X expression in WT TSCs may be more susceptible to derepression upon EED loss. To further explore this relationship, we ranked all 338 X-linked genes with allelic information by percent paternal-X expression in WT TSCs and divided this ranked list into five quintiles, each containing 67–68 genes (Fig. 5d). We then evaluated the range of percent paternal-X expression for each quintile, and identified the number of derepressed genes in each group (Fig. 5d, e). Through this analysis, we found that the derepressed X-linked genes are unevenly distributed between the quintiles (Fig. 5d, e, *p* = 0.0011, Fisher’s exact test). The derepressed genes are under-represented in quintile 1, which encompassed genes in the range of 0–1% paternal-X expression in WT TSCs and included seven derepressed genes (10% of genes in quintile 1), but are over-represented in quintile 4, which harbors genes that display 3–6% paternal-X expression in WT TSCs and included 25 derepressed genes (37% of genes in quintile 4). Quintile 4 therefore includes genes that are most susceptible to transcriptional upregulation upon EED loss, suggesting that 3–6% paternal-X expression achieves a threshold that predisposed the genes to induction in *Eed* ^*–/–*^ TSCs. Notably, these genes likely produce full-length transcripts, since in these genes enrichment of H3K36me3, a marker of transcriptional elongation [63], extended into the gene body (Fig. 5b). Quintile 5 is enriched for genes that escape X-inactivation (≥10% paternal-X expression), most of which are not subject to derepression in *Eed* ^–/–^ TSCs since they normally harbor lower H3K27me3 occupancy and thus may not be dependent on PRC2 for restriction of transcriptional activity (Fig. 5a).

Notably, although derepressed genes are over-represented in quintile 4 not all genes displaying 3–6% paternal-X expression in WT TSCs are derepressed in *Eed* ^–/–^ TSCs. Although the ratio of paternal:total X-chromosomal expression in WT TSCs is similar between the derepressed and non-derepressed genes, differences in the chromatin architecture suggested that the genes that are derepressed in *Eed* ^–/–^ TSCs may be transcribed from the paternal-X more highly in absolute terms compared with genes that are non-derepressed in WT TSCs. We therefore compared paternal-X expression level in WT TSCs for all derepressed and non-derepressed genes showing 3–6% expression in *Eed* ^–/–^ TSCs (quintile 4). The median value of paternal-X expression was in fact higher in the derepressed genes as compared with the non-derepressed genes (Mann–Whitney U test, *p <* 0.001; Fig. 5f). We additionally assessed the median paternal-X expression for all derepressed and non-derepressed genes and found that derepressed genes show significantly higher median expression overall (*p* < 0.05; Fig. 5f). Thus, the more highly transcribed paternal X-linked genes are the most sensitive to derepression upon loss of EED.

## Discussion

In this study, we defined the mode by which PRC2 propagates X-linked gene silencing by comprehensively analyzing transcription and chromatin features in imprinted X-inactivation. Imprinted X-inactivation offers a unique opportunity to investigate transcriptional silencing. Since the paternal X chromosome is exclusively inactivated in cells that undergo imprinted X-inactivation, strain-specific SNPs can be exploited to comprehensively identify genes that are silenced (or expressed) from the inactive X chromosome by RNA-seq.

### *Eed*^–/–^ TSCs lack enrichment of silencing marks on the inactive X chromosome

*Eed* ^–/–^ TSCs lose H3K27me3 and Xist RNA coating on the previously inactivated paternal-X (this study and ref. [35]), suggesting that PRC2 induces Xist RNA expression. The Xist promoter transiently undergoes heterochromatinization prior to Xist induction and X-inactivation in differentiating *XX* ESCs [28, 64]. This heterochromatic state is characterized by H4 hypoacetylation, a reduction in H3K4me2, and, notably, an increase in H3K27me3 [64]. The marking of the Xist chromatin in this manner may be necessary for Xist RNA expression, thus explaining why the absence of EED and H3K27me3 abrogates induction of Xist RNA. In addition to the loss of H3K27me3 and Xist RNA accumulation, the inactive-X in *Eed* ^–/–^ TSCs has previously been shown to be devoid of enrichment of other chromatin modifications and histone variants that normally decorate the inactive-X chromosome, including Polycomb repressive complex 1 (PRC1)-catalyzed histone H2A ubiquitylation and macroH2A [35].

### Limited X-inactivation defect in *Eed*^–/–^ TSCs

Despite the loss of both H3K27me3 and Xist RNA expression in *Eed* ^–/–^ TSCs, only 19% displayed significant loss of silencing on the paternal X chromosome in *Eed* ^–/–^ TSCs. These genes are distributed across the X chromosome, with no obvious linear clustering. The derepressed genes also do not appear be associated with the 3D conformation of the X chromosome. Moreover, X-linked genes that are enriched for H3K27me3 and Xist RNA in WT TSCs were not preferentially derepressed in *Eed* ^–/–^ TSCs, suggesting that H3K27me3 levels and Xist RNA occupancy do not predict which target loci will be derepressed upon PRC2 loss.

### Derepressed X-linked genes in *Eed*^–/–^ TSCs are transcribed and have open/active chromatin architecture in WT TSCs

**Fig. 6:**
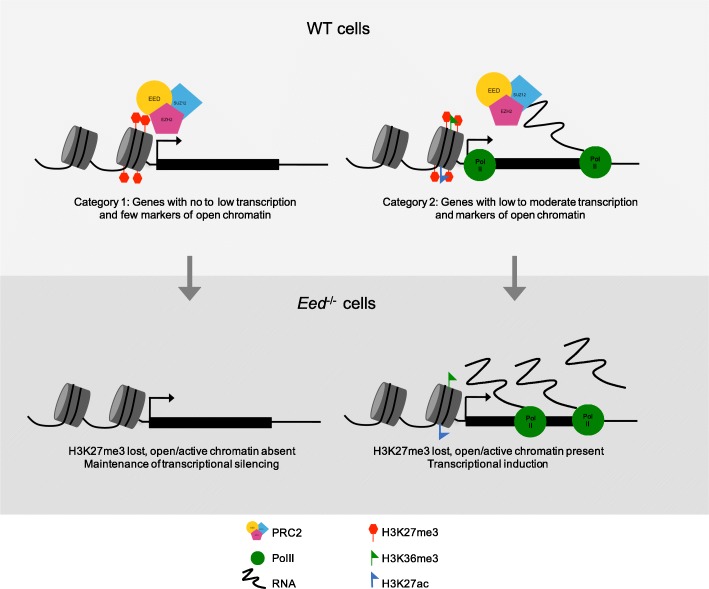
A model for PRC2 function in repressing genes. Two classes of X-linked genes are distinguished by chromatin architecture. One gene set is characterized by little to no expression from the paternal-X in WT cells and harbors only H3K27me3 at the TSSs. Upon loss of EED, these genes maintain a relatively closed chromatin conformation and are not predisposed to activation. A second class of genes is characterized in WT cells by RNA PolII occupancy and open chromatin architecture and low-level transcription. Upon loss of EED and H3K27me3, the open chromatin architecture and RNA PolII occupancy facilitate rapid upregulation of these genes

Strikingly, the level of expression in WT TSCs sensitizes the inactive X-linked genes to derepression in *Eed* ^–/–^ TSCs: genes displaying 3–6% paternal:total X-chromosomal expression in WT TSCs are particularly prone to derepression in *Eed* ^–/–^ TSCs. In this range of paternal-X expression, the genes subject to derepression upon EED loss are typically transcribed at a higher level in absolute terms compared with genes that remain repressed. In WT TSCs, the median total expression level of the genes that are derepressed is significantly higher than genes that are non-derepressed in *Eed* ^–/–^ TSCs. Thus, only the genes that reach a critical threshold of transcription from the inactive X chromosome require PRC2 to stably maintain silencing. In agreement with their higher transcription, genes that are derepressed in *Eed* ^–/–^ TSCs show a trend of increased marks of active transcription and open chromatin compared with non-derepressed genes in WT TSCs. Specifically, RNA PolII occupancy and H3K27ac exhibit higher average occupancy at genes subject to derepression upon EED loss. These derepressed genes also often exhibit H3K36me3 enrichment in WT TSCs. These hallmarks of active transcription are unexpectedly enriched, alone or in combination, on the same allele as the repressive H3K27me3 mark on the inactive-X and predict which inactive X-linked genes require continued PRC2 presence to remain repressed (Fig. 6). The open/active chromatin architecture of genes subject to derepression suggests that the TSSs of these genes are enriched for sequence specific transcription factors, which underlie the susceptibility to derepression upon PRC2 loss. PRC2 therefore serves as a brake on the induction of genes with an open chromatin and active transcriptional state.

### Bivalency-independent active chromatin at genes subject to derepression

Based on the overlap of the repressive H3K27me3 mark with features of active/open chromatin at TSSs, the derepressed genes in *Eed* ^–/–^ TSCs may belong to the “bivalent” class of genes originally identified in ESCs and in early embryos. Bivalent genes harbor both H3K27me3 and the H3K4me2/3 marks associated with active chromatin [65–68]. Marking by H3K4me2/3 is thought to induce the bivalent genes upon loss of EED/PRC2 in ESCs [65]. Similarly, bivalency has been put forth as the reason why genes in differentiating adult mouse intestinal stem cells are derepressed in the absence of EED [69]. In contrast, we find that bivalency does not predict derepression of X-linked genes in *Eed* ^–/–^ TSCs. In WT TSCs, the genes that are derepressed in *Eed* ^–/–^ TSCs are not enriched for H3K4me2 as compared with non-derepressed genes. Our data suggest that bivalency may be an imperfect proxy of open/transcriptionally active chromatin configuration. Rather than H3K4me2/3, we propose that enrichment of RNA PolII, H3K27ac, and H3K36me3 predicts which PRC2-target genes need PRC2 for repression broadly. In agreement, in WT ESCs bivalent genes that are highly susceptible to induction upon EED loss display low levels of productive transcription and DNAseI hypersensitivity.

### A novel role for EED in regulation of X-linked gene expression

Our previous analysis of *Eed* ^–/–^ TSCs concluded that EED is required to maintain silencing of genes on the inactive-X only upon differentiation of trophoblast progenitor cells in vivo and in TSCs in vitro [35]. The earlier study assessed expression of a limited number of X-linked genes and therefore may have missed the X-inactivation defects in undifferentiated TSCs. In agreement with our data herein, a recent report has detected leaky expression in WT TSCs of some inactive X-linked genes that coincides with decreased deposition of H3K27me3 [70]. Intriguingly, the loss of dosage compensation of the derepressed genes in the WT or *Eed* ^–/–^ TSCs is not catastrophic, since both sets of TSCs are viable and are able to proliferate. TSCs may therefore be insensitive to the dosage imbalance between the sexes of the subset of derepressed X-linked genes. Though dosage compensation of these genes is not required in the undifferentiated TSCs, their dose may need to be balanced later during differentiation of the trophoblast lineage. Consistent with a differentiation-dependent requirement for correct dosage compensation, *Eed* ^–/–^ TSCs are blocked in differentiation [35].

X-linked gene silencing defects observed in *Eed* ^–/–^ TSCs may also characterize random X-inactivation. A comprehensive analysis of the X-chromosome transcriptome in *Eed* ^–/–^ epiblast lineage by allele-specific RNA-seq, however, may reveal a gene-specific requirement for EED in random X-inactivation much as in imprinted X-inactivation. Whereas the analysis of expression from the inactive-X in a randomly inactivated population of cells is not directly feasible by high-throughput approaches, sequencing of single cells or of a population of cells in which random X-inactivation has been biased may be a viable strategy [39, 45–48]. If one of the two X chromosomes is preferentially inactivated in all of the cells that would normally be randomly inactivated, then allele-specific RNA-seq can be exploited to catalog gene expression defects across the X chromosome, including in an *Eed* ^–/–^ background.

## Conclusion

Despite the loss of the gene silencing elements Xist RNA and H3K27me3, only a small subset of genes on the inactive-X is derepressed in *Eed* ^–/–^ TSCs. The genes that are derepressed are characterized by elevated expression from and open chromatin features on the inactive X chromosome in WT cells. In sum, our findings reveal a unique mechanism by which PRC2 represses gene expression. Instead of ensuring complete silencing, PRC2 forestalls induction of inactive X-linked genes that are actively transcribed and characterized by specific open/active chromatin architecture, a mode of PRC2 function that may apply broadly.

## Methods

### Mice

Mice harboring a conditional mutation in *Eed* were generated by the University of Michigan Transgenic Animal Model Core using *Eed* ^tm1a (EUCOMM)Wtsi^ targeted ESCs (EUCOMM). Briefly, the ESCs were injected into blastocysts and implanted into pseudopregnant females. Males with high percentages of chimerism were bred and assessed for germline transmission. In the targeted ESCs, FRT sites flank a ß-Geo-pA cassette, which was removed by breeding *Eed* ^tm1a (EUCOMM)Wtsi^ mice with mice expressing FLP recombinase to generate the *Eed* ^fl/fl^ allele [71]. A *Mus molossinus* JF1 X chromosome was introgressed to generate *Eed* ^fl/fl^;*X* ^JF1^ *Y* males, which were bred to *Mus musculus Eed* ^fl/fl^ females that were backcrossed onto the 129/S1 background to derive TSC lines.

### TSC derivation and culture

TSCs were derived and cultured as previously described [35, 43].

### Generating stable *Eed*^–/–^ TSCs

The F1 hybrid *Eed* ^–/–^ TSC lines were derived by first plating the F1 hybrid *Eed* ^fl/fl^ TSCs at a 1:24-1:48 dilution into six-well dishes pre-seeded with mouse embryonic fibroblasts (MEFs) and allowed to adhere until the following day. The *Eed* ^fl/fl^ TSCs were then transduced with Ad5-CMV-Cre (Adenovirus type 5, University of Michigan Viral Vector Core adenoviral construct, 4 × 10^12^ particles/mL) at a multiplicity of infection (MOI) of 1000. Once cell colonies were large enough, they were subcloned into 96-well dishes pre-seeded with MEFs and re-transduced the next day with Adeno-Cre at MOI of 1000. After splitting into six-well dishes with pre-seeded MEFs, the TSCs were transduced again the next day. A portion of each 96-well samples was lysed for DNA genotyping to assess the efficiency of Cre-mediated deletion of the *Eed* floxed alleles. Subcloning, transduction, and genotyping procedures were repeated until a pure population of *Eed* ^–/–^ TSCs was derived. The non-hybrid *Eed* ^–/–^ TSC line was derived from a *Eed* ^fl/fl^ TSC line harboring a tamoxifen-inducible *Cre* transgene [Tg(UBC-cre/ERT2)1Ejb/1 J; Jax # 70001]. The *Eed* ^fl/fl^ TSCs were cultured in standard TSC media containing 6–9 ug/mL 4-hydroxytamoxifen (Sigma, #H7904) to generate *Eed* ^–/–^ TSCs. *Eed* ^–/–^ TSCs were then maintained in culture in standard TSC medium supplemented with FGF4 and heparin.

### Immunofluorescence (IF), RNA FISH, and DNA FISH

IF, RNA FISH, and DNA FISH were performed as described in detail previously [33, 72–75].

### RNA-seq sample preparation

Total RNA was isolated from TRIzol (Life Technologies, #15596-018) according to the manufacturer’s instructions and submitted to the University of Michigan DNA Sequencing Core for Poly-A purification and strand-specific library preparation using the Illumina TruSeq Library preparation kit (Illumina, #RS-122-2103). Libraries were sequenced on the Illumina HiSeq2000 platform to generate 100 bp paired-end reads.

### Mapping of RNA-seq reads

For allele-specific analysis of X-linked gene expression, we identified 413,974 SNPs on the X chromosome based on previously published whole-genome sequencing of the parental strains used to generate the TSCs [40, 42]. SNP data from whole-genome sequencing of the 129/S1 and JF1/Ms mouse strains were substituted into the mm9 mouse reference genome build (C57BL/6 J) using VCFtools to generate in silico 129/S1 and JF1/Ms genomes. Sequencing reads were separately mapped to each of the two in silico genomes using STAR [76], allowing 0 mismatches in mapped reads to ensure allele-specific mapping of SNP-containing reads to only one strain-specific genome. STAR was selected for read mapping, in part due to the improved ability to handle structural variability and indels, with the goal of reducing mapping bias to the genome most similar to the reference genome; STAR is a spliced aligner capable of detecting structural variations, and is able to handle small insertions and deletions during read mapping. STAR additionally permits soft-clipping of reads during mapping, trimming the ends of long reads that cannot be perfectly mapped (see Additional file 1: Figure S2a–c). This function would permit clipping of reads that end near indels, thus preserving mappability at SNPs near indels. To test for mapping bias, RNA-seq data from TSC lines generated from both the initial and reciprocal crosses were compared. The WT and *Eed* ^–/–^ TSC lines comprise the initial cross and harbor a *M. musculus*-derived maternal X and a *M. molossinus*-derived paternal X. The reciprocal cross contains a *M. molossinus*-derived maternal X and a *M. musculus*-derived paternal X (E.M. and S.K., in prep). To test mapping variability, we assessed the variation in allelic expression across individual SNPs in the Xist gene; we found that variation between individual SNPs was minimal, suggesting robust mapping (Additional file 1: Figure S2d). The normalized reads overlapping SNPs in maternal (active) or paternal (inactive) X chromosomes of both the initial and reciprocal cross WT TSCs were quantified and did not display a significant difference (Additional file 1: Figure S2e). In the normalized RNA-seq data, pairwise comparisons of the average SNP coverage per gene on the active X chromosome in three initial cross WT TSC lines (*Eed* ^*+/+*^) with the three reciprocal cross WT TSC lines were also very highly correlated (r > 0.91) (Additional file 1: Figure S2f). The variability in mapping to the active-X between the initial and reciprocal cross TSCs was similar to the mapping of the active-X within the initial cross TSC lines (r > 0.94) (Additional file 1: Figure S2f). These data suggest that the variability due to mapping bias between the two genomes is minimal. Although small biases may affect allelic mapping at a subset of SNP sites within a gene, the effect is mitigated since most genes contain multiple SNPs (average eight SNPs/gene; see Fig. 3a).

### Allele-specific analysis of RNA-seq data

For allelic expression analysis, only RNA-seq reads overlapping known SNP sites that differ between the 129/S1 and JF1/Ms genomes were retained. All multi-mapping reads were excluded from the analysis. For each SNP site, reads mapping to the 129/S1 and JF1/Ms X chromosomes were counted and the proportion of reads from each X chromosome identified. Allelic expression was calculated individually for each SNP site; for genes containing multiple SNPs, the paternal-X percentage for all SNPs was averaged to calculate gene-level allelic expression.

### Differential expression analysis

For differential expression analysis of paternal-X expression, all reads were first merged into a single alignment file and the number of reads per RefSeq annotated gene was counted using HTSeq. To calculate paternal-X expression for DESeq2 analysis, the total read counts from HTSeq were normalized by library size, then the number of mapped reads for each gene was multiplied by the proportion of SNP-containing reads mapping to the paternal X chromosome, calculating $\left\{\mathrm{total}\kern0.5em \mathrm{reads}\kern0.5em \times \kern0.5em \left(\frac{\mathrm{paternal}\kern0.5em \mathrm{reads}}{\mathrm{maternnal}\kern0.5em \mathrm{reads}\kern0.5em +\kern0.5em \mathrm{paternal}\kern0.5em \mathrm{reads}}\right)\right\}$. Differential expression analysis for the paternal-X expression level was then carried out using DESeq2.

### Reverse transcriptase polymerase chain reaction

Total RNA was isolated from TRIzol following the manufacturer’s instructions, then Poly-A+ selected using DynaBeads mRNA Direct kit (Life Technologies, #61012). SuperScript III One-Step RT-PCR Kit with Platinum *Taq* enzyme mixture (Life Technologies, #12574-035) was used to prepare and amplify the complementary DNA (cDNA). Primer sequences and SNP information for each amplicon are included in Additional file 6: Table S5. Amplified cDNAs were run on agarose gels and purified using the Clontech NucleoSpin Kit (Clontech, #740609). The purified cDNAs were then Sanger sequenced and sequencing traces were examined for SNPs characteristic of the *M. molossinus*-derived X chromosome and the *M. musculus*-derived X chromosome.

### Quantitative RT-PCR

Total RNA was isolated from TRIzol following the manufacturer’s instructions. DNase treatment of total RNA was performed using DNA-free DNA Removal Kit (Ambion, #1906) following the manufacturer’s instructions. A total of 0.5 μg of DNaseI-treated total RNA was used for cDNA synthesis using SuperScript III First-Strand synthesis system (Life Technologies, #18080) using a gene-specific reverse primer. A total of 1 μl (Xist or TBP) or 5 μl (Tsix) of cDNA was used in the qPCR reaction using SYBR FAST qPCR kit (Kapa Biosystems, #KK4600). Primer sequences are described in Additional file 6: Table S5.

### PCR

For DNA isolation, cell pellets from TSCs were lysed in buffer composed of 50 mM KCl, 10 mM Tris-Cl (pH 8.3), 2.5 mM MgCl_2_, 0.1 mg/mL gelatin, 0.45% NP-40, and 0.45% Tween-20. Cells in lysis buffer were incubated at 50 °C overnight, then stored at 4 °C until use. Genomic PCR reactions were carried out in ChromaTaq buffer (Denville Scientific) with 1.5 mM MgCl_2_ using RadiantTaq DNA polymerase (Alkali Scientific, #C109). Primer sequences and SNPs are described in Additional file 6: Table S5.

### X-chromosome paint

Fixed and permeabilized TSCs on coverslips were denatured in a pre-warmed solution of 2X SSC/70% formamide at 95 °C, as with DNA FISH (above). Samples were immediately dehydrated through a –20 °C ethanol series (70%, 85%, 95%, and 100% ethanol) for 2 min each followed by a final drying period for 15 min at room temperature. The coverslips with dried cells were inverted onto a glass slide with 8 μL of XCyting X-chromosome Paint (Metasystems, #D-1420-050-Fl). The cells and probe were then denatured together for an additional 2 min at 75 °C. The cells were then hybridized overnight at 37 °C in a humid chamber. Hybridized samples were washed once for 2 min at 75 °C in 0.4X SSC, pH7, with a 1:250,000 dilution of DAPI, then once for 30 s in 2X SSC with 0.2% Tergitol. Samples were then rinsed briefly with ddH_2_0 to prevent formation of salt crystals and allowed to air dry briefly. Dried samples were mounted on glass slides with Vectashield and sealed with clear nail polish.

### Microscopy

Stained samples were imaged using a Nikon Eclipse TiE inverted microscope with a Photometrics CCD camera. The images were deconvolved and uniformly processed using NIS-Elements software.

### Profiling of chromatin modifications

DNAseI-seq data and ChIP-seq data for H3K27me3, RNA PolII, H3K27ac, H3K4me2, and H3K36me3 were sourced from a study of F1 hybrid TSCs by Calabrese et al. [39], as part of GEO dataset GSE39406. RNA PolII ChIP was performed with an antibody detecting an N-terminal epitope, thus detecting both elongating and paused RNA polymerase. Allele-specific read calls were summed for each SNP identified between strains and the percent of total inactive-X reads calculated. These SNP percent values were then cross-referenced with library-size normalized Wiggle tracks for each modification based on peak calling using the MACs algorithm [39]. For regions containing SNPs, the proportion of inactive-X expression of a given peak was calculated based on the percentage of inactive X-specific reads overlapping SNP sites within the peak. ChIP-seq data assigned to the inactive-X were subdivided into 50-bp bins along the span of the X chromosome. Allelic ChIP-seq peak depth was calculated by averaging the 50-bp bins into 500-bp sections spanning from 10 kb upstream to 10 kb downstream of the TSSs of genes.

### Analysis of Xist RNA binding profiles

Xist RNA binding data were sourced from a CHART-seq study by Simon et al. [61], as part of GEO dataset GSE48649. Bedgraph tracks for allelic Xist binding were subdivided into 50-bp bins along the span of the X chromosome. CHART-seq read depth surrounding the TSSs of genes was calculated by averaging the 50-bp bins into 500-bp sections spanning from 10 kb upstream to 10 kb downstream of the TSS of each gene. CHART-seq values for individual genes were then averaged for each bin. CHART-seq read depth within gene bodies was calculated by averaging 50-bp bins into 40 bins of even length spanning the TSS to the transcription termination site of individual genes. CHART-seq values for individual genes were then averaged for each bin.

### Analysis of evolutionary strata

X-chromosome gene coordinates from all exons of the Ensembl mouse annotation for the mm9 genome build were superimposed onto the human genome (hg19 build) using UCSCs LiftOver tool. For mouse X-chromosome genes with coordinates mapping to the human X chromosome, gene locations were plotted based on the start coordinate of both mouse and human genes. Genes were assigned to evolutionary strata based on human coordinates, with stratum boundaries defined based on previously published analysis of the Y chromosome [59].

## Additional files

Additional file 1: Supplementary **Figures S1**–**S9.** (PDF 2.87 mb)

Additional file 2: **Table S1.** Allele-specific read counts for genes assayed by RNA FISH. (PDF 112 kb)

Additional file 3: **Table S2.** Paternal allele expression for X-chromosome genes with ≥ 10x coverage at SNP sites. (XLSX 96 kb)

Additional file 4: **Table S3.** Differential expression results for calculated paternal-X expression between WT and *Eed* ^–/–^ TSCs. (XLSX 79 kb)

Additional file 5: **Table S4.** Paternal allele expression for X-chromosome genes with ≥ 5x coverage at SNP sites. (XLSX 113 kb)

Additional file 6: **Table S5.** SNP locations and primer sequences for RT-PCR, Genomic PCR, and qRT-PCR amplicons. (PDF 63 kb)
