# Supplementary material for: PRC2 represses transcribed genes on the imprinted inactive X chromosome in mice
Source: Genome Biol. 2017 May 3;18:82. doi: 10.1186/s13059-017-1211-5 (PMC5415793; doi:10.1186/s13059-017-1211-5)
Supplement: Supplementary file 1 — Supplementary Figures S1–S9. (PDF 2.87 mb) [file 13059_2017_1211_MOESM1_ESM.pdf]

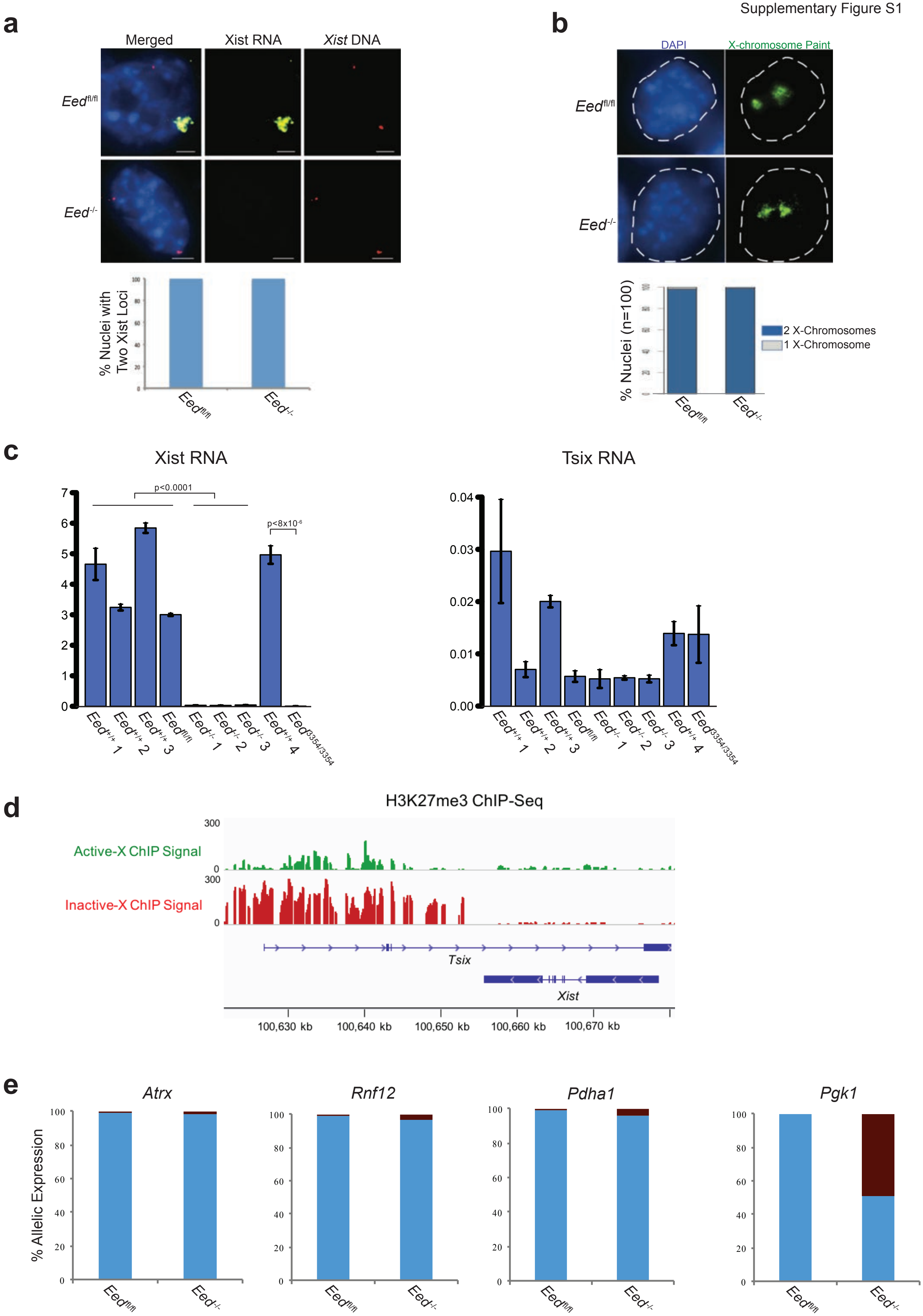
a
Merged
Xist RNA
Xist DNA
Eed fl/fl
Eed -/-
% Nuclei with Two Xist Loci
Eed fl/fl
Eed -/-
b
DAPI
X-chromosome Paint
Eed fl/fl
Eed -/-
% Nuclei (n=100)
2 X-Chromosomes
1 X-Chromosome
c
Xist RNA
p<0.0001
p<8x10-6
Tsix RNA
Eed +/+ 1
Eed +/+ 2
Eed +/+ 3
Eed fl/fl
Eed -/- 1
Eed -/- 2
Eed -/- 3
Eed +/+ 4
Eed 3354/3354
d
H3K27me3 ChIP-Seq
Active-X ChIP Signal
Inactive-X ChIP Signal
Tsix
Xist
100,630 kb
100,640 kb
100,650 kb
100,660 kb
100,670 kb
e
Atrx
Rnf12
Pdha1
Pgk1
% Allelic Expression
Eed fl/fl
Eed -/-

Figure S1 Characterization of Xist RNA expression in *Eed*$^{-/-}$ TSCs. **a** Combined Xist RNA FISH and *Xist* DNA FISH demonstrates two *Xist* loci in *Eed*$^{-/-}$ TSCs. Xist RNA is detected in green and Xist DNA in red (pinpoints). Nuclei are stained blue with DAPI. Scale bar, 2 µm. Bottom, quantification of Xist DNA FISH data. **b** Detection of X-chromosomes via X-chromosome paint (DNA FISH) demonstrates two X-chromosomes in *Eed*$^{-/-}$ TSC lines. Top, representative nucleus from each genotype. Bottom, quantification of X-chromosome number. n = 100 nuclei/sample. **c** Quantitative RT-PCR measurement of Xist and Tsix RNAs in the indicated TSCs. Y-axis represents 2^dCt for Xist/Tsix normalized to TBP. Error bars represent the standard deviation from 3 replicates. The *Eed*$^{3354/3354}$ TSCs were derived as part of Kalantry et al. (2006) [35]. **d** Enrichment of H3K27me3 at *Xist* promoter region on the inactive paternal-X in WT TSCs. **e** RNA FISH analysis of X-linked gene expression in non-hybrid *Eed*$^{fl/fl}$ and *Eed*$^{-/-}$ TSC lines as for the F1 hybrid TSC lines in Fig. 2. This *Eed*$^{fl/fl}$ TSC line harbors a tamoxifen-inducible CRE transgene (*Ubc-Cre-ERT2*) and gave rise to the *Eed*$^{-/-}$ TSC line (See Methods for details). 100 nuclei counted/gene/TSC sample.

a

b

c

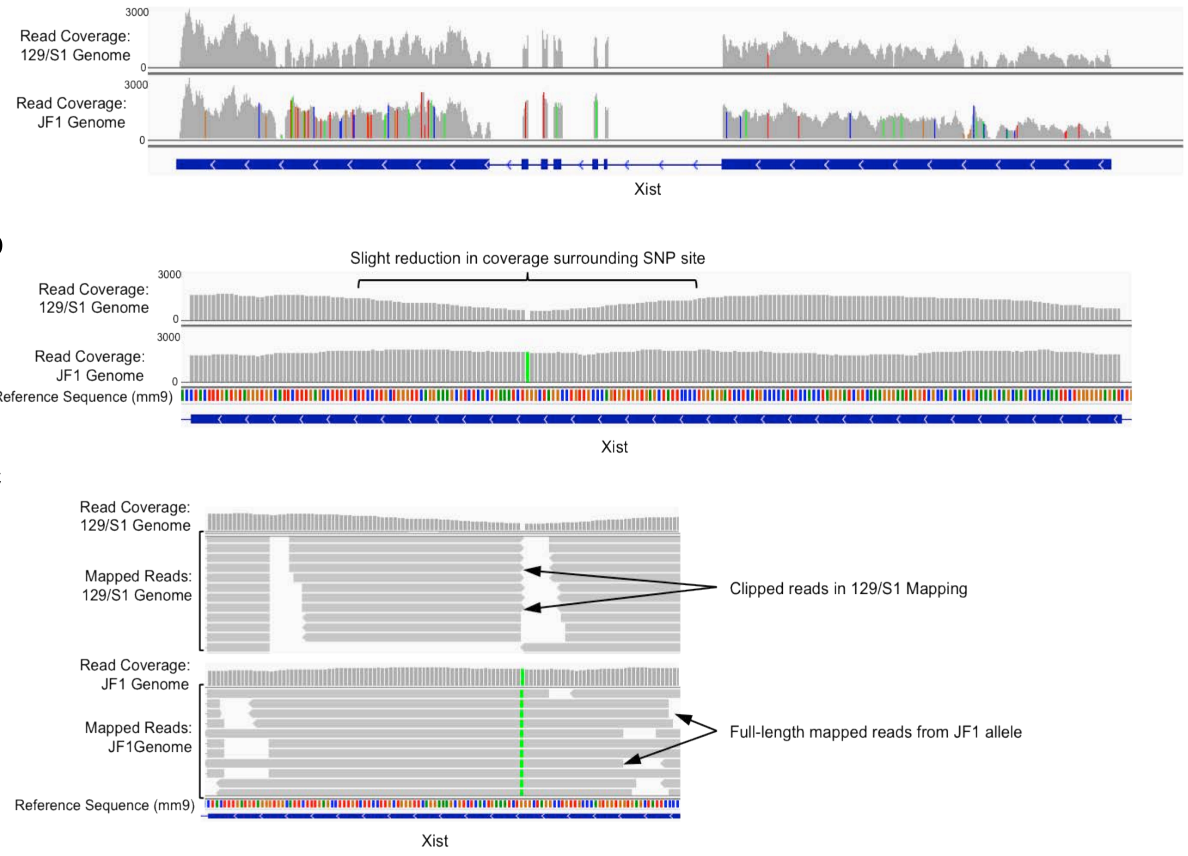

d

Allelic Expression and Inter-SNP Variability in *Xist* RNA			
**TSC Line**	**Average % Paternal-X Expression (from total reads)**	**Average % Paternal-X Expression (averaged across individual SNPs)**	**Variance Between Paternal-X Expression for Individual SNPs**
Eed$^{+/+}$1	98.7%	98.7%	0.004
Eed$^{+/+}$2	98.5%	98.6%	0.004
Eed$^{+/+}$3	98.4%	98.5%	0.004
Eed$^{fl/fl}$	98.2%	98.3%	0.004

e

f

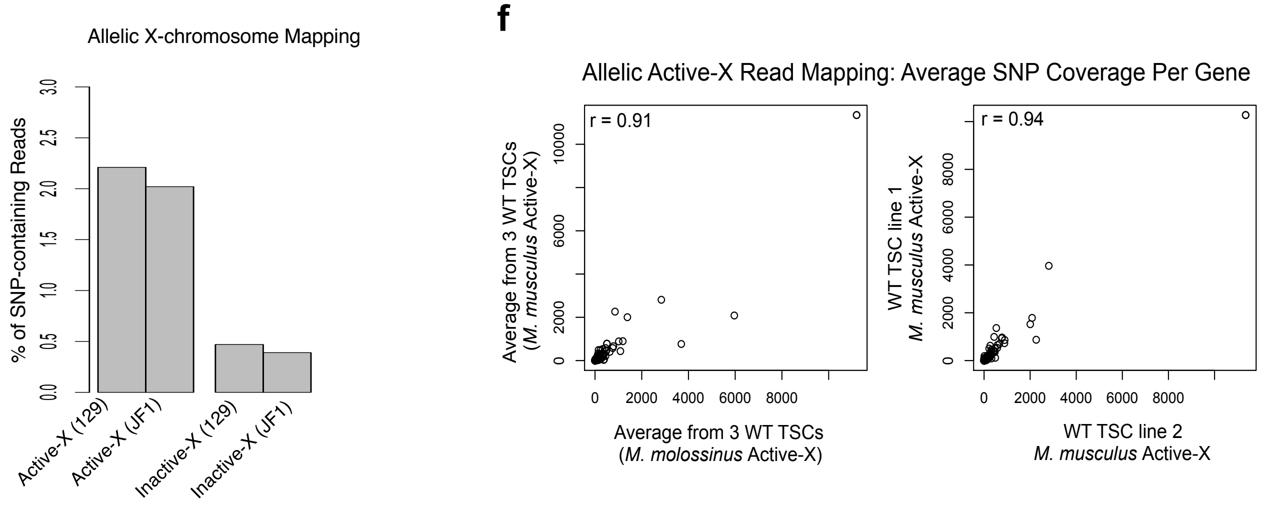

Figure S2 Allelic read mapping of RNA-Seq data with STAR. a-c Varying resolutions of reads and read coverage at the *Xist* locus from a representative WT TSC line mapped separately to the 129/S1 and JF1 *in silico* genomes. Because Xist RNA is expressed from the paternally-inherited JF1 X-chromosome, SNP-overlapping Xist RNA reads map to the JF1 allele. Colored bars indicate base pairs where the sequence of reads mapped to the 129/S1 genome (top) or JF1 genome (bottom) differs from the C57/Bl6 genome, thus marking SNP sites (**a**). At SNP sites, Xist RNA reads are reduced and do not map to the 129/S1 allele and are clipped adjacent to the SNP site (**b-c**). **d** Assessment of inter-SNP variability in the Xist RNA reads. **e** An assessment of read mapping bias to the 129/S1 vs. JF1 X-chromosomes. Left, proportion of total SNP-overlapping reads mapping to the active-X or inactive-X in reciprocal F1 hybrid TSC lines. Three lines each harboring a *M. musculus* 129/S1-derived active-X and a *M. molossinus* JF1-derived inactive-X (analyzed in this study) and three TSC lines harboring a *M. molossinus*-derived JF1 active-X and a *M. musculus*-derived 129/S1 inactive-X (E.M. and S.K., in prep) were analyzed. **f** Left, average gene-level read mapping to the active-X in reciprocal F1 hybrid TSC crosses. Y-axis, active-X mapping in three TSC lines harboring a *M. musculus* active-X and a *M. molossinus* inactive-X. X-axis, active-X mapping from the reciprocal cross. The number of normalized reads mapping to SNP sites in individual genes on the active-X genome is highly similar between the two data sets ($r = 0.91$), suggesting minimal read mapping bias. Right, analysis of active-X read mapping for two individual lines of the initial cross analyzed in this study. The active-X mapping is again highly similar between samples ($r = 0.94$). Thus, the variation in mapping to the X-chromosomes of the two strains is minimal.

a

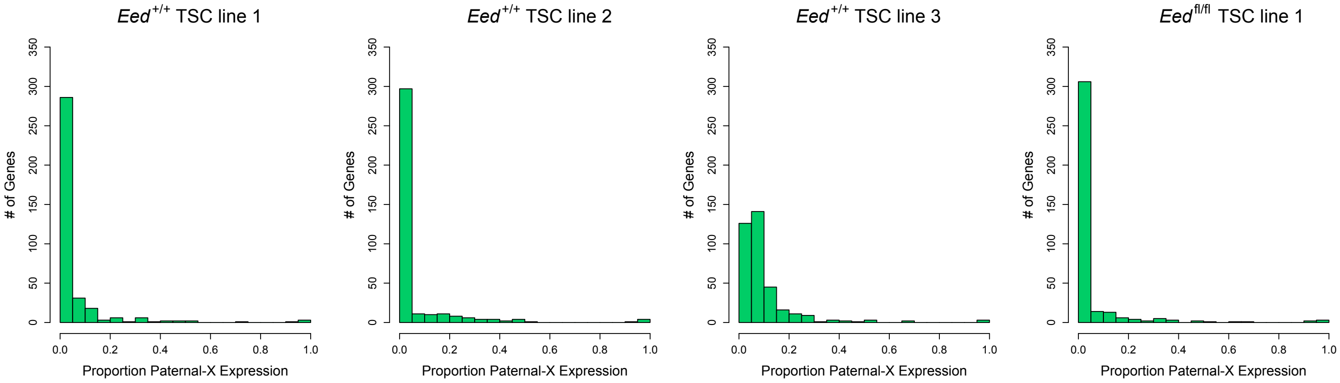
Eed+/+ TSC line 1
Eed+/+ TSC line 2
Eed+/+ TSC line 3
Eedfl/fl TSC line 1
# of Genes
Proportion Paternal-X Expression
0 50 100 150 200 250 300 350
0.0 0.2 0.4 0.6 0.8 1.0

b

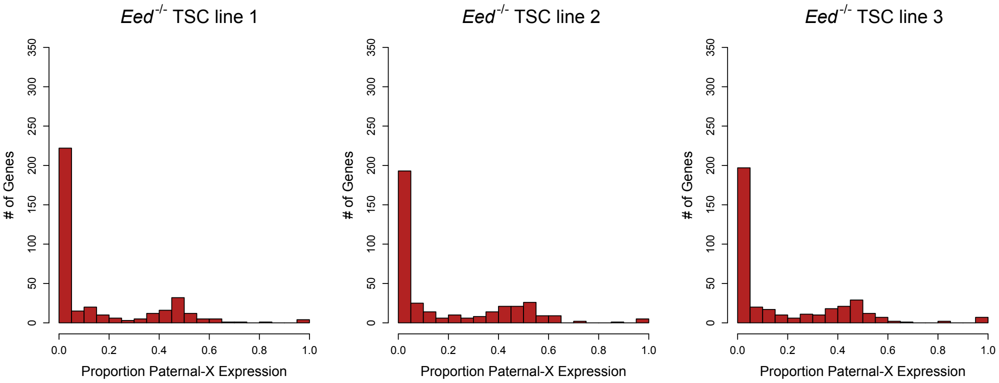
Eed-/- TSC line 1
Eed-/- TSC line 2
Eed-/- TSC line 3
# of Genes
Proportion Paternal-X Expression
0 50 100 150 200 250 300 350
0.0 0.2 0.4 0.6 0.8 1.0

Figure S3 Distribution of allelic expression profiles for X-linked genes in individual TSC lines. **a** Distribution of paternal-X expression of the 364 genes reaching the 10X coverage threshold in the four WT TSC lines. **b** Distribution of paternal-X expression of the 371 genes reaching the 10X coverage threshold in the three *Eed*$^{-/-}$ TSC lines. A greater proportion of genes in *Eed*$^{-/-}$ TSC lines show ≥10% expression from the paternal-X. For the set of genes that satisfied the 10X coverage threshold only in WT or only in *Eed*$^{-/-}$ TSCs (7% and 9% of the genes with allelic information, respectively), the ratios of paternal allele:total expression could not be compared between genotypes. For these subsets of genes, we calculated the percent paternal allele expression in the individual genotypes. Of the 26 genes that reached the 10X threshold of SNP-overlapping reads in only the WT TSC lines, only one, *Xist*, was expressed from the paternal allele. For the 33 genes that satisfied the ≥10X SNP-overlapping read coverage threshold only in *Eed*$^{-/-}$ TSCs, a higher proportion of the X-linked genes were expressed from the paternal allele (9, or 27%).

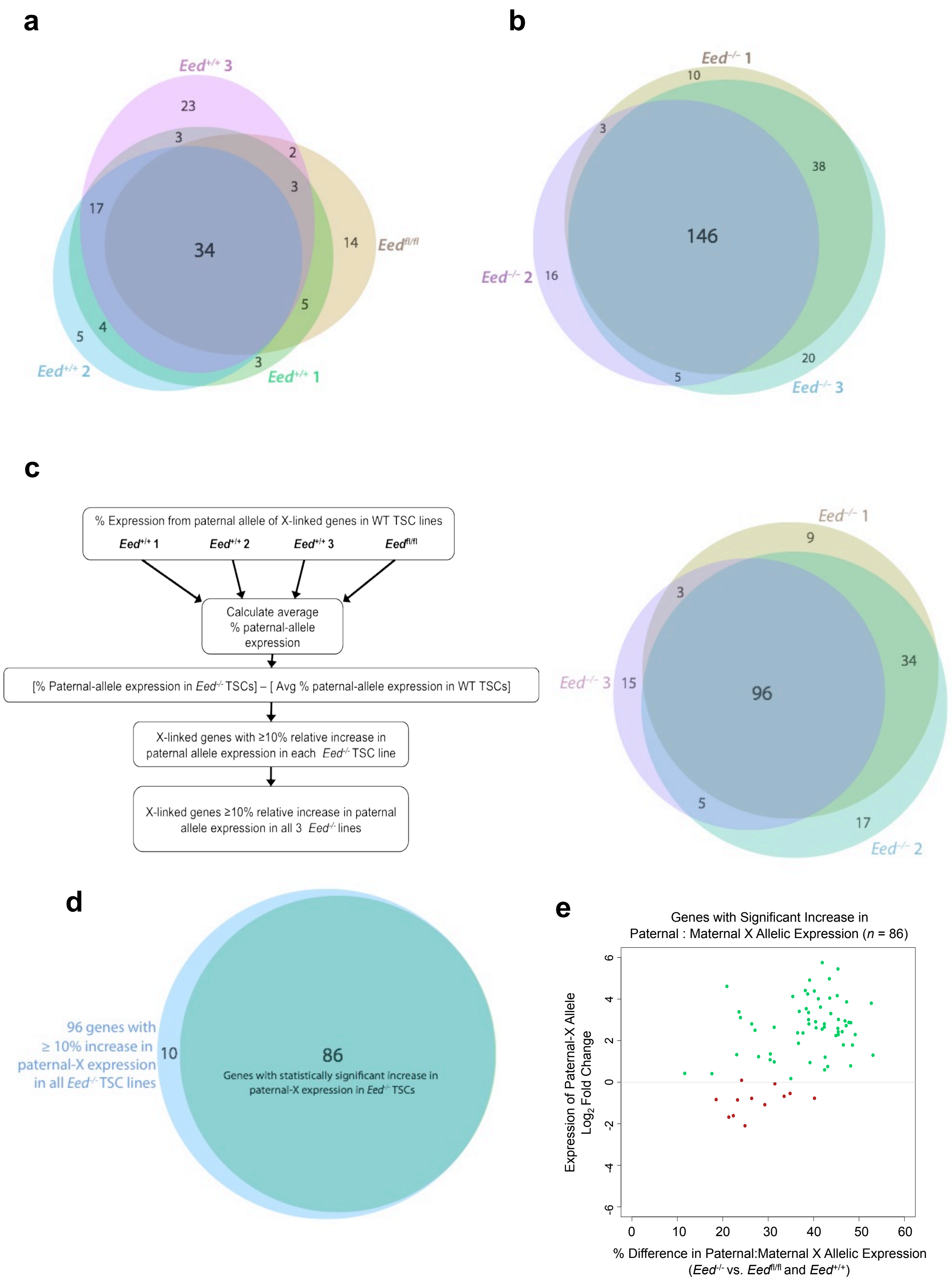
a
Eed+/+ 3
23
3
2
3
17
34
14
Eedfl/fl
5
4
5
3
Eed+/+ 2
Eed+/+ 1
b
Eed-/- 1
10
3
38
146
Eed-/- 2
16
20
5
Eed-/- 3
c
% Expression from paternal allele of X-linked genes in WT TSC lines
Eed+/+ 1
Eed+/+ 2
Eed+/+ 3
Eedfl/fl
Calculate average % paternal-allele expression
[% Paternal-allele expression in Eed-/- TSCs] – [Avg % paternal-allele expression in WT TSCs]
X-linked genes with ≥10% relative increase in paternal allele expression in each Eed-/- TSC line
X-linked genes ≥10% relative increase in paternal allele expression in all 3 Eed-/- lines
Eed-/- 1
9
3
34
Eed-/- 3
15
96
5
17
Eed-/- 2
d
96 genes with ≥ 10% increase in paternal-X expression in all Eed-/- TSC lines
10
86
Genes with statistically significant increase in paternal-X expression in Eed-/- TSCs
e
Genes with Significant Increase in Paternal : Maternal X Allelic Expression (n = 86)
Expression of Paternal-X Allele
Log2 Fold Change
6
4
2
0
-2
-4
-6
0
10
20
30
40
50
60
% Difference in Paternal:Maternal X Allelic Expression
(Eed-/- vs. Eedfl/fl and Eed+/+)

Figure S4 Identification and characterization of genes expressed from the paternal X-chromosome in WT and *Eed*$^{-/-}$ TSC lines at 5X read coverage threshold. a-b Euler diagrams assessing expression from the paternal X-chromosome totaling ≥10% of total expression in three WT *Eed*$^{+/+}$ TSC lines and one WT *Eed*$^{fl/fl}$ TSC line **(a)** and in three *Eed*$^{-/-}$ TSC lines **(b)**. **c** Comparison of percent paternal-X expression in *Eed*$^{-/-}$ TSCs compared to WT TSCs. Percent paternal-X expression for individual *Eed*$^{-/-}$ lines was compared to the average percent paternal-X expression in WT TSCs (left). **d** Identification of genes with statistically significant difference in percent paternal-X expression in *Eed*$^{-/-}$ TSCs compared to WT TSCs. Percent paternal-X expression in the four WT TSC lines and three *Eed*$^{-/-}$ TSC lines was compared by T-test and the p-values were corrected for multiple testing (Benjamini-Hochberg; FDR=0.1) as in Fig. 4. **e** Plot of $\log_2$ fold change between genotypes in the expression of paternal allele (Y-axis) versus percent difference in paternal allele expression between *Eed*$^{-/-}$ and WT TSCs (X-axis) for the 86 X-linked genes exhibiting a relative increase in the proportion of paternal:maternal allele expression, as calculated by DESeq2 [49]. Seventy-four genes are upregulated (green) and twelve genes are downregulated or show no effective change in expression (red) from the paternal-X.

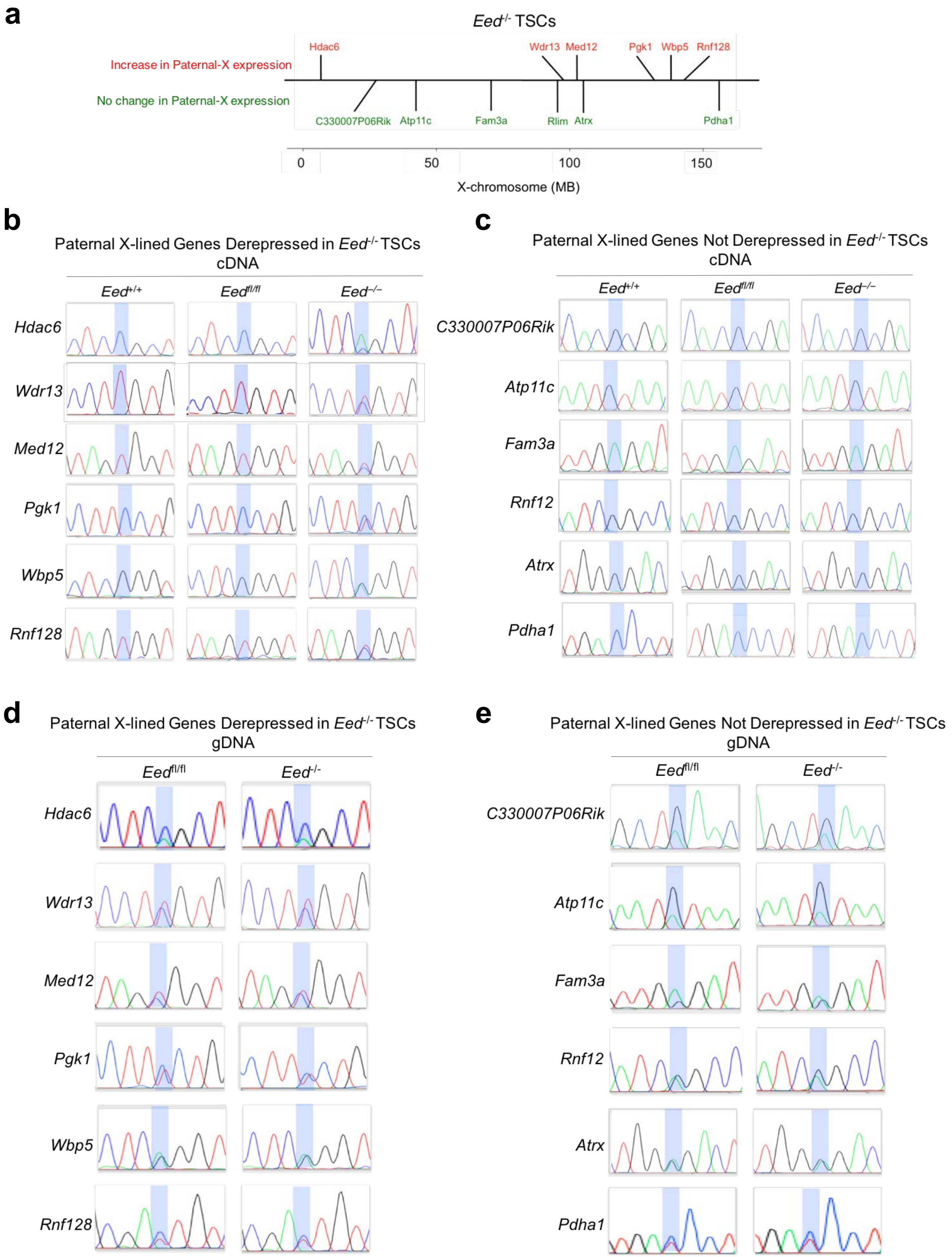
a
Eed-/- TSCs
Increase in Paternal-X expression
No change in Paternal-X expression
Hdac6
Wdr13
Med12
Pgk1
Wbp5
Rnf128
C330007P06Rik
Atp11c
Fam3a
Rlim
Atrx
Pdha1
0
50
100
150
X-chromosome (MB)
b
Paternal X-lined Genes Derepressed in Eed-/- TSCs
cDNA
Eed+/+
Eedfl/fl
Eed–/–
Hdac6
Wdr13
Med12
Pgk1
Wbp5
Rnf128
c
Paternal X-lined Genes Not Derepressed in Eed-/- TSCs
cDNA
Eed+/+
Eedfl/fl
Eed–/–
C330007P06Rik
Atp11c
Fam3a
Rnf12
Atrx
Pdha1
d
Paternal X-lined Genes Derepressed in Eed-/- TSCs
gDNA
Eedfl/fl
Eed-/-
Hdac6
Wdr13
Med12
Pgk1
Wbp5
Rnf128
e
Paternal X-lined Genes Not Derepressed in Eed-/- TSCs
gDNA
Eedfl/fl
Eed-/-
C330007P06Rik
Atp11c
Fam3a
Rnf12
Atrx
Pdha1

Figure S5 Validation of RNA-Seq results by RT-PCR and Sanger sequencing. **a** Schematic of chromosomal locations of genes selected for validation from the RNA-Seq data. **b** Sanger sequencing chromatograms of amplified cDNAs from the derepressed paternal X-linked genes in *Eed*$^{-/-}$ TSC lines by RNA-Seq. Blue highlights mark SNPs that differ between the 129/S1 strain (maternally-inherited X-chromosome) and the JF1/Ms strain (paternally-inherited X-chromosome). In all cases, expression from only the maternal allele is observed in *Eed*$^{+/+}$ and *Eed*$^{fl/fl}$ TSC lines, whereas both the maternal and paternal alleles are expressed in *Eed*$^{-/-}$ TSC lines. **c** Sanger sequencing chromatograms of cDNAs from the non-derepressed genes. These six X-linked genes exhibit no change in paternal allele expression in *Eed*$^{-/-}$ TSCs by RNA-Seq. In all cases, only the maternal allele of these genes was expressed in all genotypes. **d** Sanger sequencing of DNA amplicons detects both the 129/S1 and JF1 alleles in WT and *Eed*$^{-/-}$ TSCs for the six derepressed genes assayed by RT-PCR. **e** Sanger sequencing of DNA amplicons detects both the 129/S1 and JF1 alleles in WT and *Eed*$^{-/-}$ TSCs for the six non-derepressed genes assayed by RT-PCR.

a

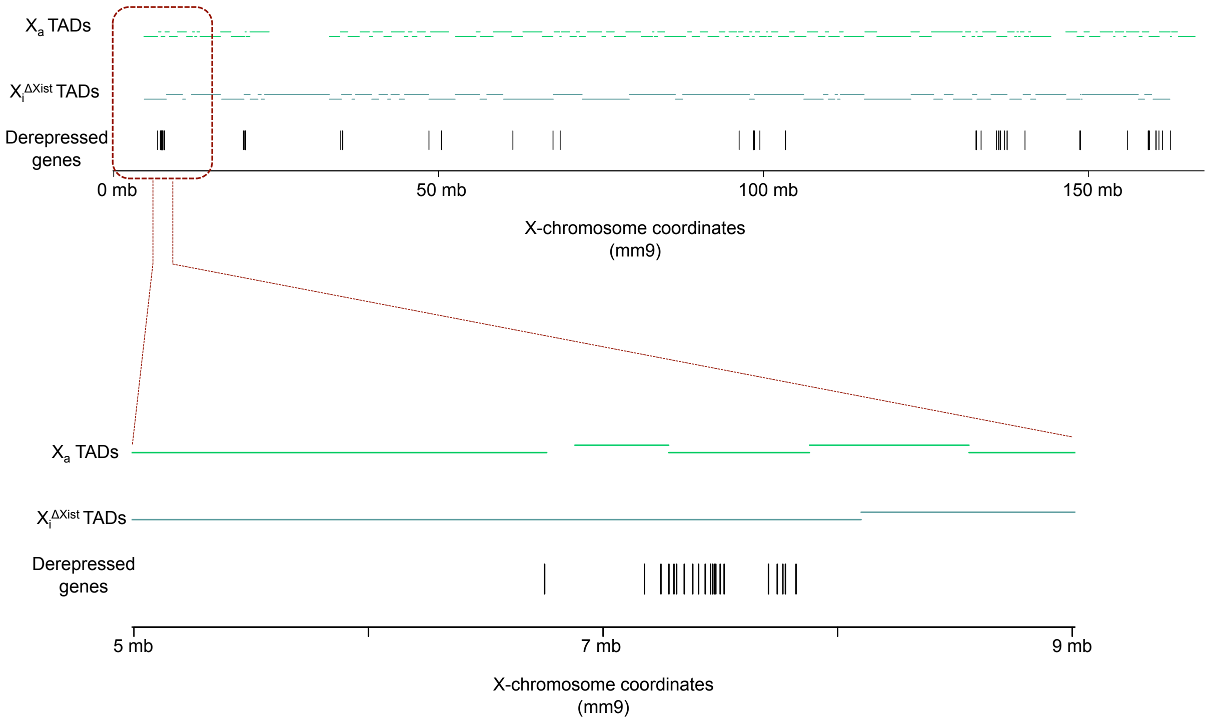
X_a TADs
$X_i^{\Delta Xist}$ TADs
Derepressed genes
0 mb
50 mb
100 mb
150 mb
X-chromosome coordinates (mm9)
X_a TADs
$X_i^{\Delta Xist}$ TADs
Derepressed genes
5 mb
7 mb
9 mb
X-chromosome coordinates (mm9)

b

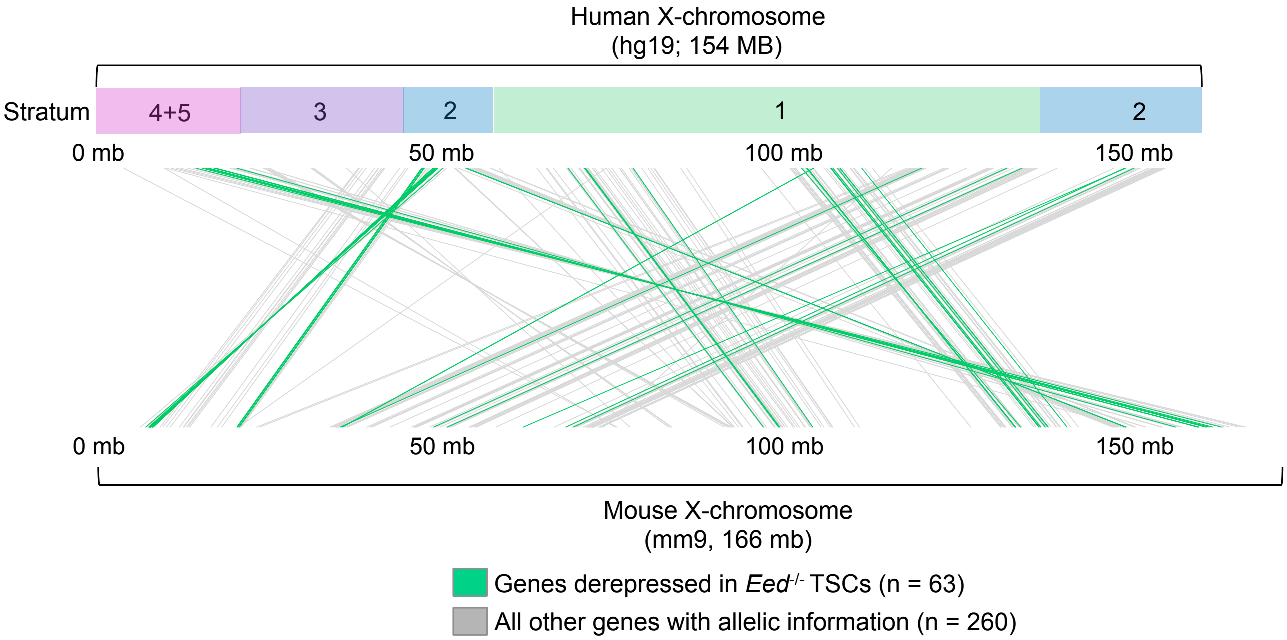
Human X-chromosome
(hg19; 154 MB)
Stratum
4+5
3
2
1
2
0 mb
50 mb
100 mb
150 mb
0 mb
50 mb
100 mb
150 mb
Mouse X-chromosome
(mm9, 166 mb)
Genes derepressed in *Eed*$^{-/-}$ TSCs (n = 63)
All other genes with allelic information (n = 260)

Figure S6 Topological structure and evolutionary stratum membership of the X-chromosome do not influence susceptibility to derepression. **a** Locations of active X-chromosome TADs and the X-linked genes derepressed in *Eed*$^{-/-}$ TSCs. Horizontal lines mark active X-chromosomal TADs [50,51]. Derepressed genes (locations indicated by vertical lines) do not cluster in individual TADs or at TAD boundaries. **b** The X- and Y- chromosomes are believed to have evolved from a pair of identical autosomes [55,56]; as the proto-X and proto-Y diverged, the proto Y-chromosome lost many of its genes. The loss of genes on the proto Y-chromosome is proposed to have driven dosage compensation on the X-chromosome in a piecemeal fashion [55,57-59]. On the human X-chromosomes, the set of genes that underwent dosage compensation at similar times during evolution, termed evolutionary strata, are collinear with map position [57-59]; the mouse X-chromosome has undergone rearrangements, but analysis of nucleotide divergence demonstrates that mouse X-chromosome genes belong to the same evolutionary strata as their human homologs [60]. To identify whether each derepressed gene is a member of one of the ancient evolutionary strata or one of the newer strata, each mouse gene coordinate for all X-linked genes with allelic information was converted to human coordinates. Lines illustrate the location of homologous human X-linked genes compared to mouse X-linked genes with an increase in paternal allele expression in *Eed*$^{-/-}$ TSCs (green lines) vs. all X-linked genes with $\geq$ 10X coverage (gray lines). Genes that require EED and/or Xist RNA for repression do not exhibit any significant clustering pattern within the evolutionary strata.

Supplementary Figure S7

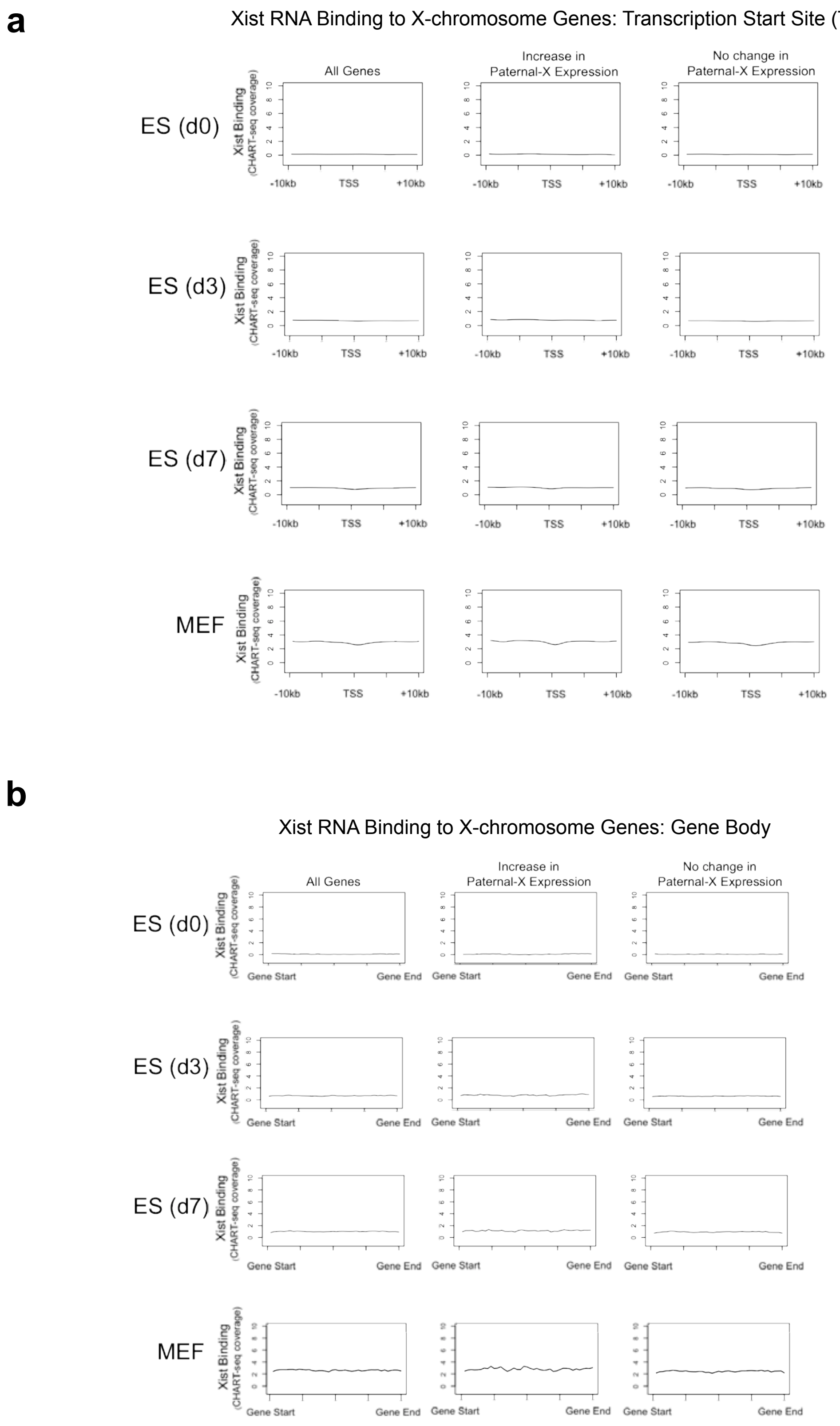

Figure S7 Xist RNA binding pattern on the inactive X-chromosome is not predictive of derepression of paternal X-linked genes in *Eed*$^{-/-}$ TSCs. a Xist RNA binding profiles 10kb upstream and 10kb downstream of TSSs in WT ESCs 0, 3, or 7 days (d) following Xist RNA induction and fully X-inactivated terminally differentiated fibroblast (MEFs), as assessed by CHART-Seq [61]. Binding patterns are shown for all genes with allelic information, including genes that are derepressed as well as non-derepressed in *Eed*$^{-/-}$ TSC lines. Genes subject to derepression do not exhibit increased Xist RNA accumulation at TSSs compared to non-derepressed genes. **b** Xist RNA binding profiles within gene bodies in ESCs 0, 3, or 7 d following Xist induction and fully X-inactivated mouse embryonic fibroblast (MEF) cells, as assessed by CHART-Seq [61]. Binding patterns are shown for all genes with allelic information, including genes that are derepressed as well as non-derepressed in *Eed*$^{-/-}$ TSC lines. Genes subject to derepression do not exhibit increased Xist RNA accumulation in their gene bodies compared to non-derepressed genes.

a

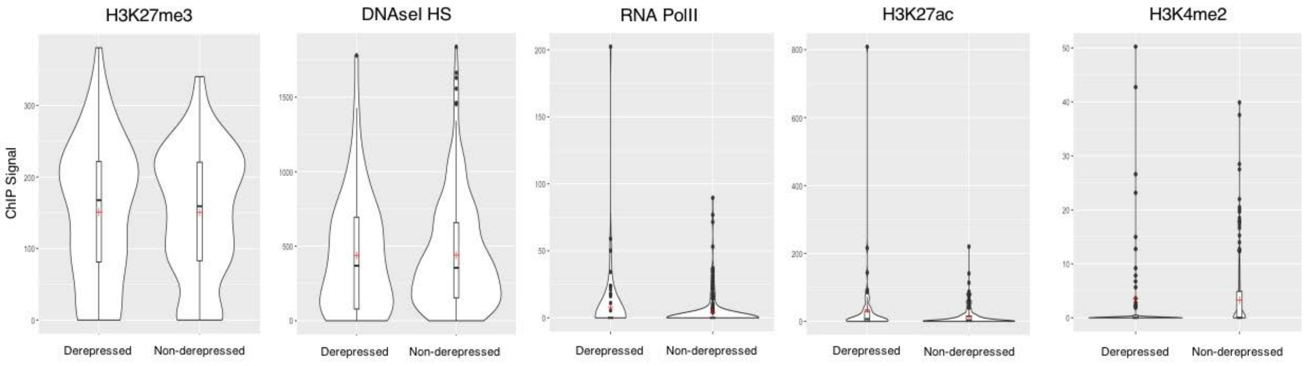

b

	Average		Number of Outliers		Proportion outliers	
	**Non-derepressed**	**Derepressed**	**Non-derepressed**	**Derepressed**	**Non-derepressed**	**Derepressed**
H3K27me3	150.3	151.0	0	0	n/a	n/a
DNaseI HS	438.4	441.0	6	1	2.7%	1.7%
RNA PolII	6.6	10.2	37	12	16.9%	20%
H3K27ac	14.1	34.9	23	5	10.5%	8.3%
H3K4me2	3.3	3.5	21	13	9.6%	21.7%

c

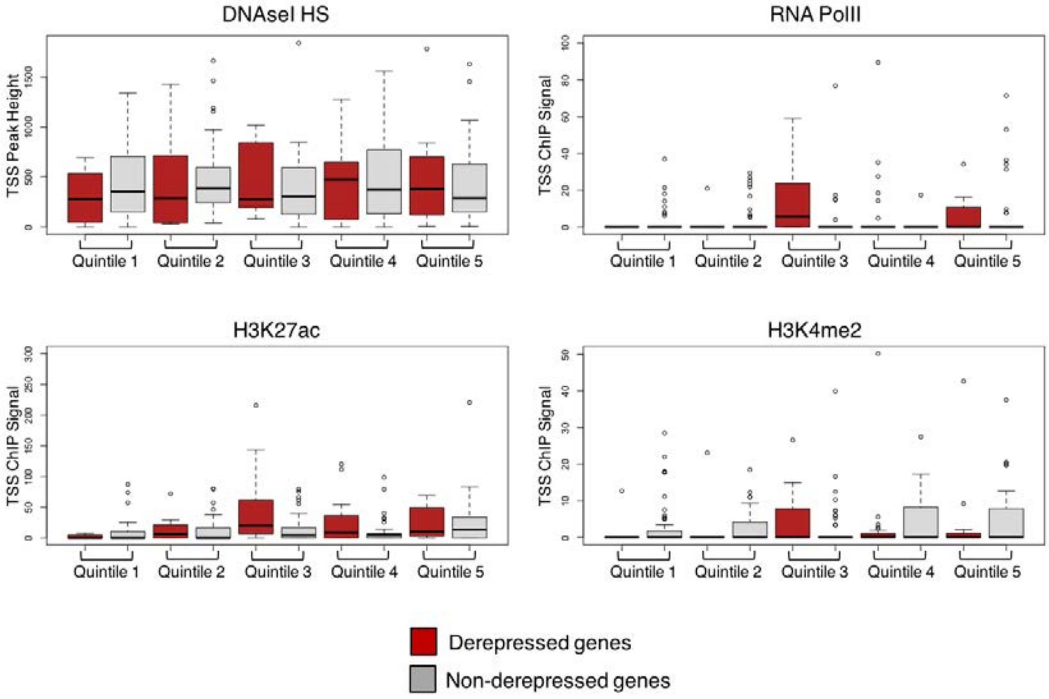

Figure S8 Chromatin profiles in WT TSCs of paternal X-linked genes that are derepressed vs. non-derepressed in *Eed*$^{-/-}$ TSCs. **a** Distribution of inactive-X profiles of DNAseI hypersensitivity (DNAseI HS), RNA PolII occupancy, H3K27ac, and H3K4me2, at the TSSs of all derepressed and non-derepressed genes with SNPs near the TSS (see Fig. 5; [39]). Horizontal black lines within central boxplots indicate the median of the distribution; red '+' signs mark the mean. **b** Summaries of average and distribution information for H3K27me3, DNAseI HS, PolII occupancy, H2K27ac, and H3K4me2 at the TSSs of derepressed and non-derepressed genes. **c** Boxplots of DNAseI HS, RNA PolII occupancy, H3K27ac, and H3K4me2 at the TSS for derepressed and non-derepressed genes in quintiles 1-5. Black lines indicate the median of the distribution.

Supplementary Figure S9

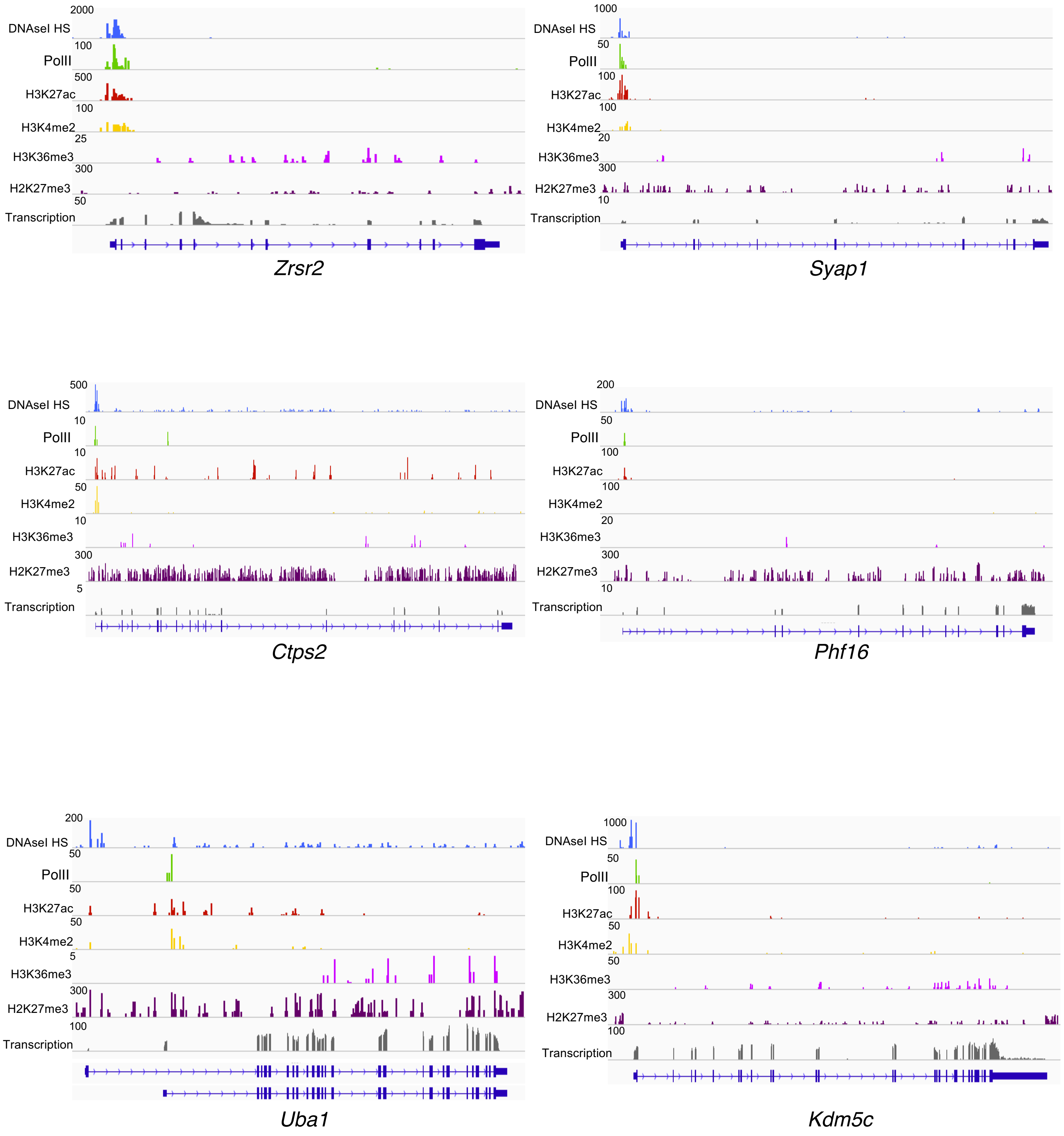

Figure S9 Example of inactive-X Chromatin Profiles for Selected Derepressed Genes. Genome browser screenshots of DNAseI HS, PolII, H3K27ac, H3K4me2, H3K36me3, and H3K27me3 occupancies together with transcription from the inactive-X alleles of five genes in WT TSCs that are derepressed in *Eed*$^{-/-}$ TSCs and of one gene that escapes X-inactivation in WT TSCs (*Kdm5c*). Allele-specific ChIP peaks were calculated based on presence of SNP-containing reads within restricted (150 bp) sliding windows of ChIP signals. In many cases, SNPs within ChIP peaks are sparse and are not always located within 150 bp of peak ChIP signal. As a result, allele-specific chromatin profiling tracks are frequently limited and choppy in coverage. The X-inactivation escapee *Kdm5c*, which is not depressed in *Eed*$^{-/-}$ TSCs, is included for comparison.
