# Supplementary material for: PRC2 represses transcribed genes on the imprinted inactive X chromosome in mice
Source: Genome Biol. 2017 May 3;18:82. doi: 10.1186/s13059-017-1211-5 (PMC5415793; doi:10.1186/s13059-017-1211-5)
Supplement: Supplementary file 2 — Table S1. Allele-specific read counts for genes assayed by RNA FISH. (PDF 112 kb) [file 13059_2017_1211_MOESM2_ESM.pdf]

Supplementary Table S1: Allele-Specific Read Counts for Genes Assayed by RNA FISH					
Gene ID	TSC Line	Total 129 reads	Total JF1 reads	% Paternal-X expression (total reads)	% Paternal-X expression (Averaged across individual SNPs)
Atrx	*Eed*$^{+/+}$ 1	5228	3	0.1%	0.1%
	Eed$^{+/+}$ 2	6338	2	0.0%	0.0%
	Eed$^{+/+}$ 3	6005	466	7.2%	7.2%
	Eed$^{fl/fl}$	5370	385	6.7%	7.0%
	Eed$^{-/-}$ 1	2710	187	6.5%	6.6%
	Eed$^{-/-}$ 2	766	53	6.5%	6.8%
	Eed$^{-/-}$ 3	1147	84	6.8%	7.3%
Rnf12	*Eed*$^{+/+}$ 1	8169	20	0.2%	0.2%
	Eed$^{+/+}$ 2	12193	30	0.2%	0.3%
	Eed$^{+/+}$ 3	11478	653	5.4%	5.4%
	Eed$^{fl/fl}$	6640	49	0.7%	0.7%
	Eed$^{-/-}$ 1	6528	187	2.8%	2.8%
	Eed$^{-/-}$ 2	5540	182	3.2%	3.2%
	Eed$^{-/-}$ 3	3965	188	4.5%	4.6%
Pdha1	*Eed*$^{+/+}$ 1	3369	5	0.1%	0.2%
	Eed$^{+/+}$ 2	2725	4	0.1%	0.1%
	Eed$^{+/+}$ 3	2492	317	11.3%	11.5%
	Eed$^{fl/fl}$	1542	31	2.0%	2.1%
	Eed$^{-/-}$ 1	2852	10	0.3%	0.3%
	Eed$^{-/-}$ 2	1988	8	0.4%	0.3%
	Eed$^{-/-}$ 3	1574	16	1.0%	1.0%
Pgk1	*Eed*$^{+/+}$ 1	2794	9	0.3%	0.3%
	Eed$^{+/+}$ 2	6816	13	0.2%	0.1%
	Eed$^{+/+}$ 3	2380	274	10.3%	10.7%
	Eed$^{fl/fl}$	3018	91	2.9%	2.8%
	Eed$^{-/-}$ 1	1007	1107	52.4%	49.7%
	Eed$^{-/-}$ 2	1053	1297	55.2%	54.8%
	Eed$^{-/-}$ 3	2403	2640	52.3%	51.8%
