# Supplementary material for: PRC2 represses transcribed genes on the imprinted inactive X chromosome in mice
Source: Genome Biol. 2017 May 3;18:82. doi: 10.1186/s13059-017-1211-5 (PMC5415793; doi:10.1186/s13059-017-1211-5)
Supplement: Supplementary file 6 — Table S5. SNP locations and primer sequences for RT-PCR, Genomic PCR, and qRT-PCR amplicons. (PDF 63 kb) [file 13059_2017_1211_MOESM6_ESM.pdf]

## Supplementary Table S5: SNP Locations and Primer Sequences for RT-PCR, Genomic PCR, and qRT-PCR Amplicons.

| Gene Name | SNP Location | 129 Allele | JF1 Allele | RT-PCR Primer (Forward) | RT-PCR Primer (Reverse) | Genomic PCR Primer (Forward) | Genomic PCR Primer (Reverse) |
|---|---|---|---|---|---|---|---|
| Hdac6 | chrX:7524355 | A | C | GGCGGACTAGAAAGAGCCT | CCCTTGAAGCCCCACAACTA | CCGGTCTGGCGGACTAGAA | GAAGGGGTGACTGGGGATTG |
| Wdr13 | chrX:7706230 | A | G | AGTGGACGCGAGTTTCGG | CCCACCACAGTGAGGTTGTT | TGTTGACTGGCTGGAAGGTG | TCCCACAAATGGCTCCTTGG |
| Med12 | chrX:98488083 | A | G | AGGTTCACCAACTGTTGCCA | GCTGCTGCCGGTATACAGAT | CCATCAACAGCACCACTGTC | CTTTTTGCGCTCCTCAGTGC |
| Pgk1 | chrX:103393283 | C | T | GAAGGGAAGGGAAAAGATGC | TGTGCCAATCTCCATGTTGT | CCATGGTGGGTGTGAATCTGC | TAAACTGTCCTCAGTTACCCCAT |
| Wbp5 | chrX:132780961 | T | C | TTTGTACCGCACCAGGCTAA | GCTTCGGTTAGCCTTCCAAC | TCATTGCATTCCCGTATTCTGC | GGATGGCTTCGGTTAGCCTT |
| Rnf128 | chrX:136199996 | G | A | TGTGGACCCGTGGCTTTTAG | TAAGCACCTGGAGACAAACCC | TGGTAAAGCCAATTCCTACCCC | GCATAGCCAGATGTGTGGTC |
| C330007P06Rik | chrX:34403690 | G | A | GGAGCTATGCCGAAAGTCGT | GCTTCCAATTCAGCCAACTCC | CCGCAACCCAACAGTGAACTA | TGGTAATTCTCCTTCCAGGAGTG |
| Atp11c | chrX:57496031 | G | A | TGAAAGGCAGCCCCATAACA | AAGATTCGGATTCCTGGCACT | TGCCATGCCCATGGAAAAATG | GCCCACTTTTCTTGTTTCCTTAC |
| Fam3a | chrX:71638049 | A | G | TCCCTACACCCAGACAACA | TCATTGACATCTCCAGCCCAC | ACAAACCAGTGACCACCCAG | CTCACGAGGAACTGAGCCTG |
| Rlim | chrX:101159081 | C | T | GAGCCCCGATGAAAATAGAGC | GGTCGGCACTTCTGTTACTGC | GCTCTGGTTCTCCGATGCTC | TGAACCATCCACTAGGCGTC |
| Atrx | chrX:103042079 | G | A | GGGATTGCTGCTGTGAGTCT | CCACCATCTTCTTGCCATCT | CTGAATCTCCCTCCACAGCC | GGGATTGCTGCTGTGAGTCT |
| Pdha1 | chrX:156562514 | G | A | GGGACGTCTGTTGAGAGAGC | GCACTTCAAAGGGAGGATCA | TAGCCCTGTGAGCCTTCAGA | GCTACCGCACTCGAGAAGAA |

| Gene Name | qRT-PCR Primer (Forward) | qRT-PCR Primer (Reverse) |
|---|---|---|
| **Xist** | CAAGAAGAAGGATTGCCTGGATTT | GCGAGGACTTGAAGAGAAGTTCTG |
| **Tsix** | CCTGCAAGCGCTACACACTT | GGAGAGCGCATGCTTGCAAT |
| **TBP** | TTCAGAGGATGCTCTAGGGAAGA | CTGTGGAGTAAGTCCTGTGCC |
